# Connectivity independent protein-structure alignment: a hierarchical approach

**DOI:** 10.1186/1471-2105-7-510

**Published:** 2006-11-21

**Authors:** Bjoern Kolbeck, Patrick May, Tobias Schmidt-Goenner, Thomas Steinke, Ernst-Walter Knapp

**Affiliations:** 1 Macromolecular Modeling Group, Institute of Chemistry and Biochemistry, FU Berlin, Takustrasse 6, 14195 Berlin, Germany; 2 Computer Science Research, Zuse Institute Berlin, Takustrasse 7, 14195 Berlin, Germany

## Abstract

**Background:**

Protein-structure alignment is a fundamental tool to study protein function, evolution and model building. In the last decade several methods for structure alignment were introduced, but most of them ignore that structurally similar proteins can share the same spatial arrangement of secondary structure elements (SSE) but differ in the underlying polypeptide chain connectivity (non-sequential SSE connectivity).

**Results:**

We perform protein-structure alignment using a two-level hierarchical approach implemented in the program GANGSTA. On the first level, pair contacts and relative orientations between SSEs (i.e. α-helices and β-strands) are maximized with a genetic algorithm (GA). On the second level residue pair contacts from the best SSE alignments are optimized. We have tested the method on visually optimized structure alignments of protein pairs (pairwise mode) and for database scans. For a given protein structure, our method is able to detect significant structural similarity of functionally important folds with non-sequential SSE connectivity. The performance for structure alignments with strictly sequential SSE connectivity is comparable to that of other structure alignment methods.

**Conclusion:**

As demonstrated for several applications, GANGSTA finds meaningful protein-structure alignments independent of the SSE connectivity. GANGSTA is able to detect structural similarity of protein folds that are assigned to different superfamilies but nevertheless possess similar structures and perform related functions, even if these proteins differ in SSE connectivity.

## Background

Protein-structure alignment remains a great challenge in structural genomics and constitutes an important tool for applications in protein classification, protein-structure prediction, drug design and studies of evolutionary relationships. The number of known three-dimensional protein structures determined by NMR spectroscopy and X-ray crystallography is increasing rapidly. At the end of the year 2005 the Protein Data Bank (PDB) [1] contained more than 33,000 protein structures. Hence, efficient methods to detect structural similarity among different proteins, analogous to methods for sequence alignment are clearly needed.

The pairwise protein-structure alignment problem can be defined as the task of identifying maximal common substructures of two proteins according to a given similarity measure. Algorithms solving this problem use different representations of protein structures. GRATH [2], SSM [3], TOP[4], TOPS [5], MATRAS [6], PROTEP [7] and VAST [8] work on protein secondary structure level only. Such secondary-structure representation is also used for index-based database searches [9,10]. DALI [11], CE [12], SSAP [13], FASE [14] and SCALI [15] work on the residue level or a combination of secondary-structure and residue level. Another approach employs methods derived from computer vision to compare 3D models [16]. TOPSCAN [17] uses topology string representations. Other approaches tackle even the problem of aligning multiple structures [18,19]. Theoretical work characterizing protein architectures focused initially on pure β-strand proteins [20-23] or on pure a-helical proteins [24]. Proteins of mixed topology containing a-helices and β-strands were considered as undirected labeled graphs [20,25-27].

Useful comparison of three-dimensional protein structures require a structure-similarity score that is transferable to biological and chemical classifications reflecting protein architectures. Several measures for protein-structure similarity have been proposed. The *root mean square deviation *(RMSD) of equivalent atom positions of a protein pair [28] is widely used. Other similarity measures are *distance map similarity *[29] and *contact map overlap *(CMO) [30,31], which employ residue pair distances and contacts, respectively. CMO is based on the notion of *contacts *between two residues. A contact map captures a 3D structure in condensed form, representing the 3D protein conformation as a symmetrical, square, Boolean matrix of *contacts*. Such contact maps are also used as basic information to elucidate protein structures from NMR spectroscopy [32]. Although they simplify the description of protein structures, they still provide all necessary information to reconstruct a protein structure with sufficiently high accuracy. In the CMO approach, the protein-structure alignment problem is solved by analyzing the similarity of their contact maps. CMO-based structure alignment was introduced by Godzik and Skolnick [30] and was proved to be NP-hard by Goldman et al. [33]. However, Caprara et al. [34] succeeded with integer programming to get solutions for maximum CMO of protein-structure pairs in reasonable CPU times. Nevertheless, the protein-structure alignment problem is computationally hard to solve.

To reduce the computational burden of protein-structure alignment connected with direct use of pairwise-residue assignment, we employ in the present study a hierarchical approach. On the first level of the hierarchy, protein-structure alignment of SSEs is performed. On the second level, solutions for the CMO are searched on the residue level. In analogy to protein sequence alignment, structure alignment methods can work with either a global or a local strategy. Global strategies start from whole structures and remove poorly matched parts of the structure. In contrast, local strategies start from small matching units and attempt to enlarge and merge these. The presented method (GANGSTA: Genetic Algorithm for Non-sequential, Gapped protein STructure Alignment) uses a global strategy.

Protein architectures are essentially defined by the spatial arrangement of α-helices and β-strands (SSEs). These SSEs generally form the central part of protein structures, while loop, turn and coil structures are more irregular and preferentially localized on the protein surfaces. Furthermore, the composition and arrangement of a-helices and β-strands are highly conserved evolutionary in contrast to the conformations of loops, turns and coils connecting these SSEs. Hence, restriction to these SSEs is advantageous for structure comparison, since it focuses on the regular parts of the structure, which can be characterized more compactly, thereby facilitates recognition of structural similarity. GANGSTA considers only these regularly structured SSEs, which greatly reduces the complexity of the protein-structure alignment problem and facilitates structure alignments with non-sequential SSE connectivity.

It is a widely assumed that similar protein structures can be aligned while the SSE connectivity in the polypeptide chain (sequential SSE connectivity) is conserved. Nevertheless, a considerable number of proteins possess different SSE connectivity but share the same architecture (i.e. the same spatial arrangement of SSEs: see Yuan et al. [15] for a detailed list). It has been shown that permuted SSE alignments (i.e. alignments with non-sequential SSE connectivity) occur often [35]. Structure alignments involving proteins of different SSE connectivities pose a computational challenge. Using protein representations in terms of graphs on the secondary-structure level, we can describe structure alignment as a search for the maximum common subgraph [7,20,26], a problem that is known to be NP-complete. Therefore, we decided to use a genetic algorithm (GA) to perform connectivity-independent alignments on the SSE level, since evolutionary algorithms provide reasonable strategies to solve NP-complete problems [36]. GAs have been used previously for structural alignment [37-40] and for detecting appropriate structure templates in homology modeling [41]. Only few methods, such as SARF [42,43], K2 [37,38], MASS [19,44] or SCALI [15], can align protein-structure fragments in non-sequential order. However, none of these methods optimizes the matching of protein graphs.

GANGSTA was developed to produce high quality global protein-structure alignments independent of SSE connectivity by optimizing the contact map overlap. The method can be used for pairwise protein-structure alignment or fast database searches with respect to a specific protein structure and is available through a web server [45]. For the case of pairwise structure alignment, we provide a statistical significance related to our similarity measure in the form of a *P*-value, the probability that a better score can be reached by structure alignment of unrelated proteins. The performance of GANGSTA was assessed in pairwise structure alignments and database scans with sequential and non-sequential SSE connectivity. We show GANGSTA's ability to detect structural similarity of protein folds that are assigned to different superfamilies but nevertheless posses similar structures and perform related functions, even if these proteins differ in SSE connectivity.

## Results

### Protein-structure alignment with GANGSTA: an example

To demonstrate the capability of GANGSTA to find protein structures with different SSE connectivities, we consider the structure alignment of the two SCOP domains 2uagA1 and 1gkuB1. In CATH [46] these protein domains correspond to 2uagA01 and 1gkuB02, respectively. The name convention of protein domains in SCOP and CATH are as follows: {pdb_id|CHAIN_id|domain_id} for instance {2uag|A|01}. They share the same protein-structure class (alpha/beta) but belong to different fold and superfamily categories in SCOP. Both structures have an incomplete Rossmann structure motif [47] in common. The Rossmann structure motif is ubiquitous in the universe of protein structures. It occurs with different SSE connectivities and comprises four α-helices and four β-strands. In the incomplete Rossmann structure motif one dangling α-helix is missing. Generally, it serves as a device for binding functionally relevant cofactors, such as nucleotide di(tri)phosphates and flavins.

In the SCOP classification scheme [48], the polypeptides 2uagA1 and 1gkuB1 belong to the folds "MurCD N-terminal domain" and "P-loop containing nucleoside triphosphate hydrolase", respectively. In CATH [46], these two polypeptides are classified in the homologous superfamilies "NAD(P)-binding with Rossmann-like domain" and "P-loop containing nucleoside triphosphate hydrolase", respectively. Both proteins share the same level of CATH topology "Rossmann-fold".

**Figure 1:**
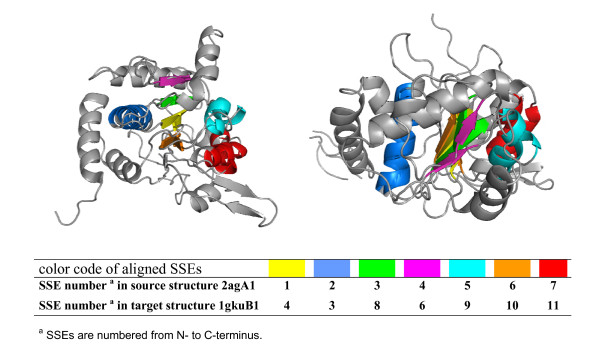
**GANGSTA structure alignment of protein domains 2uagA1 and 1gkuB1**. The aligned structures are displayed in two different orientations. Aligned SSEs of source (2uagA1) and target (1gkuB1) structures have the same color. The SSEs connecting loops and SSEs not considered for the alignment are displayed in light gray in both structures. The figure was created with PyMol [68].

In the pairwise structure alignment mode the smaller protein structure (source) is superimposed on the larger protein structure (target). In the target structure only the SSEs useful for the alignment are considered, while the omission of an SSE in the source structure (introducing a gap) is penalized (see method section). Fig. 1 shows the result of the GANGSTA structure alignment for the two polypeptides as superposition of aligned SSEs. Table 1 summarizes results obtained from the pairwise structure alignment of the complete set of SSEs of source structure 2uagA1 on the target structure 1gkuB1. Although the two protein domains possess different SSE connectivities, GANGSTA was able to align them with a good *P*-value (below 0.05 corresponding to a confidence level of 95%, see methods section) considering all SSEs of the source structure (i.e. introducing no SSE gaps).

**Table 1:** Summary of structure alignment of 2uagA1 (source) and 1gkuB1

quantity	value	comments and details
qresst MathType@MTEF@5@5@+=feaafiart1ev1aaatCvAUfKttLearuWrP9MDH5MBPbIqV92AaeXatLxBI9gBaebbnrfifHhDYfgasaacH8akY=wiFfYdH8Gipec8Eeeu0xXdbba9frFj0=OqFfea0dXdd9vqai=hGuQ8kuc9pgc9s8qqaq=dirpe0xb9q8qiLsFr0=vr0=vr0dc8meaabaqaciaacaGaaeqabaqabeGadaaakeaacqqGXbqCdaqhaaWcbaGaeeOCaiNaeeyzauMaee4CamhabaGaee4CamNaeeiDaqhaaaaa@3547@, eq. (9)	0.6310	1 is identity, 0 is minimum
RMSD(C_α_) [Å]	4.222	0.0 is identity
G-score, eq. (10)	0.0667	0.0 is identity
*P*-value	0.0085	<0.01 is significant
N_alnRes_	42	number of aligned residues
alignment topology	non-sequential	(1,2,3,4,5,6,7) → (4,3,8,6,9,10,12)^**a**^
Number of gaps, *N*_*gap*_	0	ignored SSEs of source structure

^**a **^Assignment of SSEs between source (left) and target (right) structures numbered from N- to C-terminus according STRIDE. SSE numbering is explained in Fig. 2.

### Significance of the GANGSTA score for pairwise structure alignments

**Figure 2:**
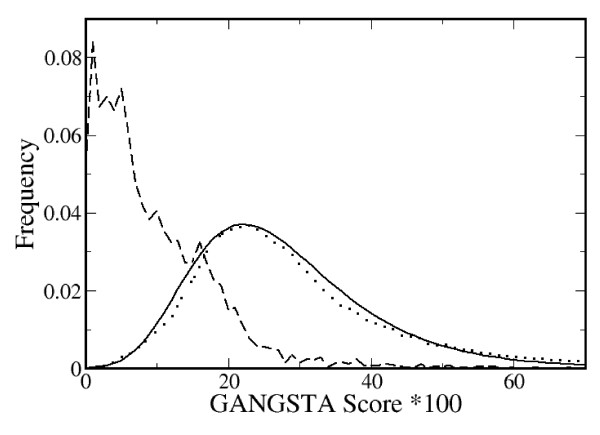
**Distribution of GANGSTA score**. The distribution of the GANGSTA score, eq. (10), for aligned protein pairs of the same (dashed line) and of different (dotted line) SCOP superfamilies. The Gumbel distribution, eq. (15), *f*(Score*100) (solid line) was fitted with *a *= 22.2013 and *b *= 9.9384. For more details see method section.

One important application of protein-structure alignment is to find out whether a pair of proteins is structurally or evolutionarily related. SCOP or CATH databases are often used for such a classification task. Whether the similarity measure employed in GANGSTA (GANGSTA score) is suitable to assign two protein structures to the same SCOP superfamily was tested by a statistical study similar to the one described in [14]. For that purpose, we performed structure alignments of 4892 protein domain pairs where both proteins belong to the same SCOP superfamily (dataset SAME_SF40) and 88909 structure alignments of domains pairs where both proteins belong to different SCOP superfamilies (dataset DIFF_SF40). The two datasets are explained in more detail in the method section. For the protein-structure alignments from both datasets the distributions of GANGSTA scores are shown in Fig. 2. A Gumbel distribution was fitted to the GANGSTA score distribution of the DIFF_SF40 dataset with mean μ = 27.938 and standard deviation σ = 12.746 [see eqs. (18) and (19)], as described in the method section. According to Fig. 2, the distributions of GANGSTA scores of the two datasets overlap partially. Hence, it is not possible to conclude reliably from the similarity of two protein structures that they belong to the same superfamily of proteins. The ability of the GANGSTA score to discriminate between related and non-related protein structures can be illustrated as *coverage *versus *error rate *plot as shown in Fig. S2 of Supplement Data [see Additional file 1] [14,49] evaluated according to Ortiz et al. [50]. In short, the *coverage *is the ratio of true positives at a given *P*-value threshold, while the *error rate *defines the number of false positives for that threshold. In the above application, GANGSTA is able to detect 48% and 67% of the SCOP superfamily relationships at a confidence level of 99% and 95%, respectively.

### GANGSTA structure alignments with non-sequential SSE connectivity

We studied the performance of GANGSTA for alignment of protein structures with non-sequential SSE connectivity that are known from literature (example: the four helix bundles or the TRAF-domain-like fold studied in [19,44]). Additionally, we show significant alignments of protein structures with non-sequential SSE connectivity involving the Rossmann and Rossmann-like structure motif according to classifications in SCOP or CATH. All comparisons were done in the pairwise structure alignment mode using Stride [51] for SSE assignment.

### Four-helix bundles

**Figure 3:**
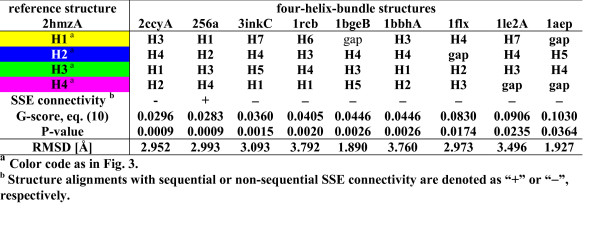
**Structure alignments for reference structure 2hmzA against the Four-Helix-Bundle dataset**. Nine structures from the Four-Helix-Bundle dataset were aligned against the reference structure 2hmzA. For each structure alignment the table lists the SSE assignment, the GANGSTA score, the *P*-value, and the RMSD. α-helices are numbered from N- to C-terminus according to SSE connectivity in the reference structure 2hmzA. The structures are ordered according *P*-value.

**Figure 4:**
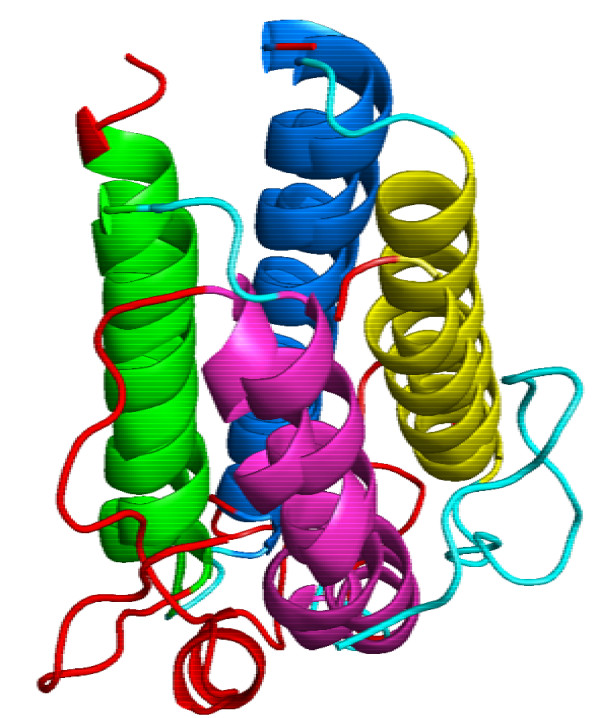
**Superposition of two aligned four-helix-bundle proteins**. The polypeptide backbone of the connecting loops and not aligned SSEs are colored cyan for 2hmzA and red for 3inkC. Aligned SSEs have the same color coding.

As a first application we selected the protein domain 2hmzA as reference structure for four-helix-bundles and aligned it pairwise with the nine other protein domains from the Four-Helix-Bundle dataset (see method section for details; results are given in Fig. 3). For all pairwise alignments the SSE assignment (relative to the reference structure), the GANGSTA score, *P*-value, and RMSD are listed. GANGSTA was able to align all structures within 95% confidence level. Only three protein domains (1le2A, 1aep, 1flx) were not aligned within 99% confidence level and they all contain alignment gaps (i.e. some SSEs of the source structure were not aligned). All structure alignments were superimposed with an RMSD smaller than 3.5 Å. It is noteworthy that only the alignment of 256a with the reference structure 2hmzA is optimal with sequential SSE connectivity. Fig. 4 shows the structural superposition of the two protein domains 2hmzA and 3inkC.

### β-strands of TRAF immunoglobin dataset

**Figure 5:**
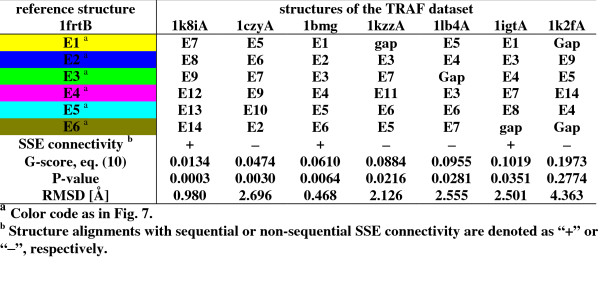
**Structure alignments for reference structure 1frtB against the TRAF dataset**. Seven structures from the TRAF dataset were aligned against the reference structure 1frtB. The SSEs are numbered from N- to C-terminus according to SSE connectivity in the reference structure 1frtB. The structures are ordered according *P*-value.

**Figure 6:**
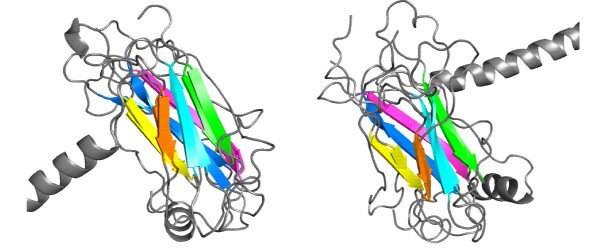
**Superposition of aligned structures of 1frtB with 1czyA (left) and with 1kzzA (right)**. Aligned SSEs have the same color coding.

We aligned the reference structure 1frtB against all other seven domains of the TRAF dataset (see method section for details; results are shown in Fig. 5). GANGSTA was able to align six of the seven proteins within 95% confidence threshold. Only protein domain 1k2fA could not be aligned with a significant *P*-value (0.2774). This is the only protein in the TRAF dataset that could be aligned to the reference structure if two gaps are introduced in the 1k2fA structure, resulting in a structure superposition with 4.3 Å RMSD. For all other structures the corresponding RMSDs are smaller than 2.7 Å. All structure alignments of 1frtB with proteins from different families possess different SSE connectivities: Only the structure alignments with members of the same family as the reference structure (1bmg, 1igtA, 1k8iA) possess the same SSE connectivity. Fig. 6 shows the superposition of 1frtB with 1czyA (left) and with 1kzzA (right), two proteins from SCOP superfamilies that differ from 1frtB. Both alignments are non-sequential in SSE connectivity relative to 1frtB.

### Rossmann structure motif

**Figure 7:**
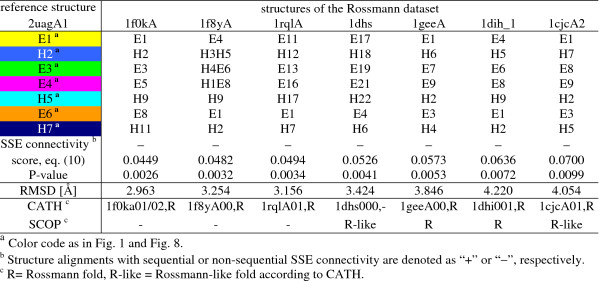
**Results of structure alignments of reference structure 2uagA1 against the structures of the Rossmann dataset**. All structures from the Rossmann dataset were aligned against 2uagA1. SSEs are numbered according to SSE connectivity of 2uagA1 from N- to C-terminus. Structures are ordered according *P*-value.

Here we consider a sufficiently complex and widespread structure motif, the Rossmann structure motif [47], which was first identified in dinucleotide-binding proteins. We used the SCOP domain 2uagA1 as reference structure and the Rossmann dataset (see method section for details) as target structures. Six of the seven proteins are classified as Rossmann-fold in CATH topology level except 1dhs, which is classified in SCOP as Rossmann-fold. The results are shown in Fig. 7. GANGSTA was able to align all proteins with the reference structure 2uagA1 within the 99% confidence level. All alignments were non-sequential with respect to the SSE connectivity of the reference structure, and all superpositions could be made with RMSD smaller than 4.2 Å.

### Robustness of GANGSTA

The following tests are made to demonstrate the robustness of GANGSTA against variations in structure and SSE connectivity for a given fold motif. These tests also demonstrate that GANGSTA can retrieve approximately the same set of target structures when different source structures of the same motif are used. This symmetric behavior is an important feature that makes GANGSTA suitable for database scans. To assess these features we performed the following tasks.

1. A database scan with 2uagA1 (incomplete Rossmann structure motif) as reference structure (source) was conducted. The results were clustered according to the SSE connectivity pattern with respect to the reference structure.

2. Two new reference structures were generated by considering only the aligned SSEs (representing the incomplete Rossmann structure motif) (reduced structures) of 1dhs and 1cjcA2. These proteins belong to the two largest clusters containing structures of different SSE connectivity (with respect to the Rossmann structure motif) that were obtained from the preceding database scan. The corresponding reduced structures were used as reference (source) structures for two additional database scans.

3. The results obtained from all three database scans (task 1 and 2) were compared to determine whether GANGSTA is able to retrieve the same set of similar structures despite variations in the reference structures.

Since GANGSTA returns all alignments for a database scan, we used a cutoff at a GANGSTA score [see eq. (10)], of 0.15. This cutoff corresponds to a *P*-value of 0.127, giving a significance level of 87% for pairwise structure alignment. Since we are using an estimated contact overlap q [eq. (9)] for database scans, this *P*-value is only an upper bound on the real structure similarity and serves only as selection criterion sufficient for this experiment.

For the first task, the top 100 structure alignments with non-sequential SSE connectivity were monitored. The last of these structures was found at rank position 154. We considered in the following all aligned structures (with sequential and non-sequential SSE connectivity) of a rank lower then or equal to 154. From this set of 154 structures, the alignments involving gaps in the source structure were omitted. This yields a total of 135 structures (result set 1) that can be aligned with a significance level of 99% or higher against the reference structure (see Table 2). These 135 protein structures were then grouped into clusters containing the same SSE connectivity as the reference structure (i.e. the incomplete Rossmann structure motif 2uagA1). This results in 44 different clusters [see Additional file 1, Table S8]. For the subsequent two tasks, we considered the largest two clusters (with 11 and 9 members, respectively) of structures with SSE connectivities different from the reference structure 2uagA1 (result set 1).

**Table 2:** Structure alignments versus 2uagA1

structure	qresst MathType@MTEF@5@5@+=feaafiart1ev1aaatCvAUfKttLearuWrP9MDH5MBPbIqV92AaeXatLxBI9gBaebbnrfifHhDYfgasaacH8akY=wiFfYdH8Gipec8Eeeu0xXdbba9frFj0=OqFfea0dXdd9vqai=hGuQ8kuc9pgc9s8qqaq=dirpe0xb9q8qiLsFr0=vr0=vr0dc8meaabaqaciaacaGaaeqabaqabeGadaaakeaacqqGXbqCdaqhaaWcbaGaeeOCaiNaeeyzauMaee4CamhabaGaee4CamNaeeiDaqhaaaaa@3547@, eq. (9)	RMSD	G-score, eq. (10)	*P*-value^**a**^	# residues^**b**^
1dhs	0.603	3.11 Å	0.0505	0.0036	42
1cjcA2	0.671	3.94 Å	0.0715	0.0106	37
average^**c**^	0.652	3.83 Å	0.0661	0.0082	n/a

Results from the database scan with reference structure 2uagA1. Structure alignment results for 1dhs and 1cjcA2, and results averaged over all 135 alignments without gaps.

^**a **^Upper bound for *P*-value.

^**b **^Number of aligned residues.

^**c **^Average statistics over all 135 structure alignments against the complete 2uagA1 structure.

**Figure 8:**
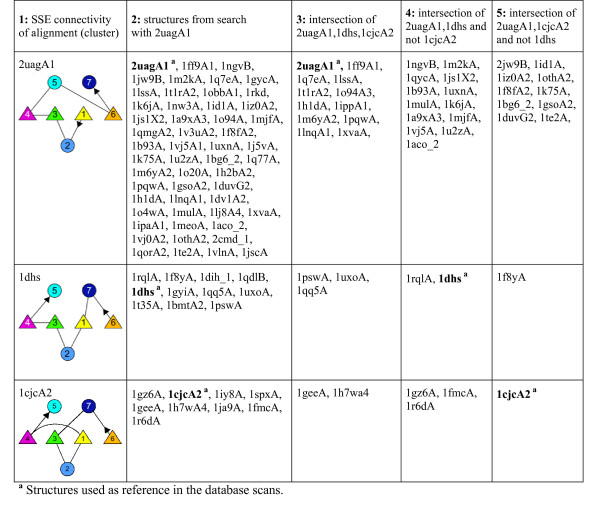
**Structures of the two largest clusters of protein domains containing a Rossmann structure motif obtained by structure alignment with 2uagA1**. Column 1 shows the SSE connectivity of clusters with respect to 2uagA1. Column 2 contains the search results for 2uagA1 found for different SSE connectivities. Column 3 lists the structures that were retrieved using both 1dhs and 1cjcA2 as reference structures (intersection of sets of aligned structures found with 2uagA1, 1dhs, 1cjcA2) considering the specified SSE connectivity only. Column 4 (5) list the intersection of those structures obtained with 2uagA1 and with reference structure 1dhs (1cjcA2) that are not contained in the set of structures obtained with 1cjcA2 (1dhs) (considering the specified SSE connectivity only).

For the second task, we chose two representative incomplete Rossmann structure motifs from the two largest clusters with non-sequential SSE connectivity: 1dhs and 1cjcA2. Since both structures are larger than 2uagA1, we reduced them to the aligned SSEs: 1dhs(98–123, 277–320,328–357) and 1cjcA2(8–26,30–37,61–71,78–100,360–370), respectively. We performed a database scan with these two reduced reference structures to obtain result sets 2 and 3. Finally (third task), we compared the structures from the two largest clusters in result set 1 (column 2 in Fig. 8) with those retrieved by alignment with either 1dhs or 1cjcA2 or with both. Fig. 8 lists the results of this assessment. From 73 structures found with 2uagA1 as reference structure we retrieved 35 with 1dhs and 29 with 1cjcA2 as reference structure (column 2 of Fig. 8).

### Protein-structure alignment tasks with sequential SSE connectivity

Most programs or servers for protein-structure alignment deal with sequential structure alignments only and most of the known curated structure alignments or benchmark sets for structure alignment are constructed to test methods preserving the sequential SSE connectivity. To obtain a more representative comparison with other alignment methods we tested the performance of GANGSTA for structure alignments with exclusively sequential SSE connectivity.

The two structure alignment tasks we conducted here complement the evaluation of web-based programs and servers for structure alignment applied in recent performance tests by Novotny et al. [52,53]. The authors identified protein structures as true positives (i.e., structures that are known to have an architecture similar to the reference structure) by using CATH classification [46]. The various servers evaluated in [53] all use different databases and scoring systems, so simple counting of true positives was not feasible. Therefore, we used a simple binary scoring system: at least one true positive either was or was not found in the list of significant hits. For servers that did not indicate the significance of the hits, up to 100 hits were examined. This was done for GANGSTA as well, see below. A true positive was defined on the topology level in the CATH classification scheme. Each reference structure was submitted to all servers evaluated in the Novotny study, and it was determined whether any of the structures, other than the reference structure, were found as true positive. To have a similar test scenario, we decided to reproduce these structure alignment tasks using the database scan version of GANGSTA. All database scans were performed using DSSP for SSE assignment. We used the GANGSTA score [eq. (10)] to rank the resulting structure alignments. However, no *P*-value could be evaluated, since for database scans GANGSTA calculates only an estimated contact map overlap *q *[eq. (9)] to increase the computational performance (see method section).

The first task was based on a selection of protein domains (Novotny dataset, see method section) belonging to four different CATH classes (*mainly*-α, *mainly*-β, *mixed *α-β, *few SSEs*) as used in [53]. Proteins from the fourth CATH class (*few SSEs*) have only low secondary structure content and thus few SSE contacts. Since GANGSTA considers α-helices and β-strands only, we tested it only on those proteins of the Novotny dataset (reduced Novotny dataset) belonging to CATH classes mainly-α, mainly-β and mixed-α-β. This resulted in 53 reference proteins [see Additional file 1, Table S5]. The results of the structure alignment with GANGSTA and 11 other methods are shown in Table 3. Except for the data obtained with GANGSTA all data were taken from the literature [52,53]. Average performances differ slightly from the literature values, since the structures with low secondary structure content were omitted. In analogy to the preceding investigations on the Novotny dataset [53] the assignment of a reference structure was successful with GANGSTA, if at least one target with appropriate CATH topology was found among the top 100 ranked protein domains. GANGSTA was able to detect true positives for 52 of all 53 reference structures (98%) of the reduced Novotny dataset except for the mainly-α protein 1c3u. This protein had been moved to another topology in more recent CATH versions [46] (Table 3 and [Additional file 1, Table S5]), so we could not compare the GANGSTA results to results listed for other methods. Hence, GANGSTA reaches the best result possible for the reduced Novotny dataset.

**Table 3:** Comparison of different structure alignment methods for three structure classes according to CATH [46]

Program/Server	Mainly-α 19 str.^**a**^	Mainly-β 19 str.^**a**^	Mixed-α-β 15 str.^**a**^	Average performance (%)
CE	17	19	13	93
DALI	14	19	14	89
DEJAVU	14	19	9	79
**GANGSTA**	**18^b^**	**19**	**15**	**98**
LOCK	0	14	11	47
MATRAS	11	19	14	83
SSM	5	13	10	53
TOP	14	18	12	83
TOPS	2	15	14	59
TOPSCAN	15	12	9	68
VAST	12	17	15	83
YAKUSA	17	19	14	94

Except for GANGSTA all data were taken from literature [52, 53]. Average performances differ slightly, since structures with low secondary structure content were omitted. The 53 proteins of the Novotny dataset (see method section) were aligned against the SCOP40 reference database. For the GANGSTA evaluation the assignment of a reference structure was successful, if at least one target with appropriate CATH topology was found among the top 100 ranked protein domains.

^**a **^Target protein structures belonging to the specified CATH class that are used for assignment to the appropriate CATH topology (for details see text).

^**b **^Since protein 1c3u was moved to another topology class in more recent CATH versions, 18 is the maximum number of correct structure alignments achievable. Thus, preventing GANGSTA from reaching the maximum performance of 100% (Table S5 [see Additional file 1]).

The second task considers a database search with eleven pairs of structures from the Fischer dataset (see method section for details) that were considered as difficult structure alignment cases [8] before. According to Novotny et al. [53], a search was considered to be successful, if for a reference structure the defined result structure or a homologous structure was found. Again true positives were searched among the top 100 ranked targets from structure alignment. GANGSTA was able to find appropriate result structures for each of the eleven protein pairs (see Table 4 for more details). Seven results were found at top 1 position, eight within the top 10, and all within the top 50 ranked structures. Hence, in this test GANGSTA outperforms DALI and CE, which both found ten out of eleven possible structure pairs [53].

**Table 4:** Results for the Fischer dataset.

**protein pair**		**successful matches**		
**reference structure**	**result structure**	**rank**	**PDB code**	**CATH level^a^**
1bgeB	2gmfA	1	1bgc	H
		2	1alu	H
		6	1lki	H
1cewI	1molA	49	1eqkA	H
		66	1stfI	H
1cid01	2rhe	25	1eajA	H
		35	1ojaE1	H
1crl	1ede	1	1llfA	S
1fxiA	1ubq	14	1m94A	H
		23	1c1yB	H
		26	1lm8B	H
		40	1lfdA	H
1ten	3hhrB	2	1fnf02	H
		4	1f6fB2	H
		5	1fhyB2	H
		8	1cd9B2	H
1tie	4fgf	1	1avwB	H
		2	1wba	H
		6	1jlxA1	H
		8	1md6A	H
		12	1q1uA	H
2azaA	1paz	1	1qhqA	H
		2	1jzgA	H
		3	1sdfA	H
		4	1plc	H
		8	1jw0A3	H
2sim	1nsbA	1	3sil	H
		14	1usrA	H
3hlaB	2rhe	1	1k5nB	H
		4	1fp5A2	H
		13	1mjaH	H
		15	1ojae2	H
1g61	1jdw	1	1jdw	result
		2	1g62A	H
		54	1bwdA	S

For all 11 Fischer pairs the structures from the database are given, which are most similar to the specified target structure together with their rank and CATH classification.

^**a **^CATH hierarchy levels [46]: H: same homologous superfamily, S: same sequence family.

### Implementation

The GANGSTA structure-alignment method is implemented in C++ in a first version only for UNIX systems. It is available as web application at [45]. The user can perform pairwise structure alignments or database searches against a library of 3D structures. The database in use is the SCOP40 (see method section, databases). The assignment of secondary structure can be done with DSSP [28], Stride [51] or according to the HELIX/SHEETS records in PDB [1] files. In Table S1 [see Additional file 1] the runtimes for some exemplary pairwise structure alignments and database searches are shown. All calculations were done on a Linux AMD Opteron 242 system, using one thread for the entire program including all initializations.

## Discussion

We have tested GANGSTA on different datasets to assess its performance for challenging tasks in protein-structure alignment. These include (1) classification of protein superfamilies, (2) searching for structure alignments with non-sequential SSE connectivity, (3) testing robustness against structural variation, and (4) comparison with other methods considering datasets of protein structures that require sequential SSE connectivity.

We could show that for structure alignments from different SCOP superfamilies the distribution of GANGSTA scores follows the well known Gumbel distribution. The same distribution was reported by Levitt and Gerstein [54], MAMMOTH [50] and FASE [14], which use different measures of structural similarity and different optimization algorithms. The discrimination between structurally related and non-related proteins (At a confidence level of 95% (99%), 67% (48%) true positives were found by GANGSTA as pictured in the coverage-error plot in Fig. S1 [see Additional file 1]) is comparable with other methods. At a confidence level of 99% PrISM [49] reported 54% and MAMMOTH [50] 50% true positives. At a confidence level of 95% MAMMOTH reported 60% and FASE [14] 72% true positives. In contrast to these studies GANGSTA reports the *P*-value for SCOP superfamily classification instead of SCOP fold classification. This test is more demanding, since protein structures may share the same SCOP fold but belong to different SCOP superfamilies.

Generally protein-structure alignments are validated using classification schemes that discriminate according to specified criteria between related and un-related structures. For this purpose most studies use the CATH or SCOP database of classified proteins. However, these databases were also generated with specific classification criteria, which naturally may build in biases. This adds to the difficulties of fairly comparing different methods of protein-structure alignment. Kolodny et al. [55] showed that comparisons based on receiver operating characteristic (ROC) curves are often unsatisfactory with respect to the quality of protein-structure alignment. So far, the best insight into the quality of a protein-structure alignments can be obtained by visual inspection. This depends on the structural and functional features upon which the viewer focuses and is obviously subjective in nature.

Protein-structure alignments from different SCOP families and superfamilies have demonstrated that GANGSTA is able to find reasonable structure alignments that may provide new insights for structure-function relationships of proteins and also for structural motifs that occur with different SSE connectivities. The results for the Rossmann dataset demonstrate that GANGSTA finds structural similarities for proteins that are known to have similar function but no obvious structural or sequence similarity. The Rossmann structure motifs are ubiquitous, appearing in the large enzyme family of kinases [56] that catalyze the transfer of phosphate groups. In these proteins, the Rossmann structure motif constitutes just a small fraction of the whole structure, which can differ significantly in the remaining part of the structure. Hence, SCOP classifies these proteins in different superfamilies, such as MurCD N-terminal domain, FAD/NAD(P)-binding domain, HAD-like, NAD(P)-binding Rossmann-fold domains, DHS-like NAD/FAD-binding domain, UDP-Glycosyltransferase/glycogen phosphorylase, and Flavodoxin-like. The structural similarity found by GANGSTA hints at functional similarity in nucleotide binding. GANGSTA is able to detect the structural similarity of those proteins despite their topological differences with respect to SSE connectivity. Protein structures with different SSE connectivity often exhibit large structural variations in terms of RMSD, but can simultaneously have large contact overlaps and a GANGSTA score [eq. (10)] close to zero, corresponding to high quality structure alignment.

In a test for robustness of GANGSTA the incomplete Rossmann structure motif 2uagA1 could be retrieved with database scans using 1dhs or 1cjcA2 as reference structure. The robustness also demonstrates the symmetric behavior of GANGSTA. From the 73 structures found with 2uagA1 from the largest three clusters (column 2 in Fig. 8) 35 (columns 3+4) and 29 (columns 3+5) were retrieved by structure alignment on database scans with the Rossmann structure motif taken from the structures 1dhs and 1cjcA2, respectively, although there are large variations in the Rossmann structure motif of these three reference structures (see RMSD in Table 2). The fact that variation in SSE connectivity did not influence the retrieval of similar structures is not surprising, since GANGSTA considers the SSEs as independent secondary structure elements and disregards the connecting polypeptide loops.

Analog to a recent study [15], GANGSTA found different clusters of protein domains with different SSE connectivities for the Rossmann structure motif. Among these aligned structures with non-sequential SSE connectivities are protein domains belonging to different CATH [46] topology levels or different SCOP [48] fold levels. Hence, GANGSTA is able recognize structure similarities of protein domains that share the same CATH architecture but belong to different CATH topologies. Thus, GANGSTA may be useful to classify protein structure domains. Rossmann structure motifs with different SSE connectivities carry out similar functions, which is a clear example of convergent evolution. The fact that protein function can be correlated with CATH architecture rather than the more detailed CATH topology is an interesting observation.

Although GANGSTA was designed and implemented specifically to find unusual protein-structure alignments with non-sequential SSE connectivity that are hard to detect, we could show that even for sequential SSE connectivity GANGSTA is able to compete with other established protein-structure alignment methods like DALI [11], VAST [8], YAKUSA [52], and CE [12]. Regarding the number of aligned residues and the overall RMSD results individual pairwise protein-structure alignments with GANGSTA are generally somewhat inferior to the results obtained with other methods. But, for the more imprecise database scan method GANGSTA outperforms structure-alignment methods that consider sequential SSE connectivity only.

## Conclusion

GANGSTA is able to find meaningful protein-structure alignments independent of the SSE connectivity. The RMSD is often used as a similarity measure for structure alignment. We could show that functionally related protein domains can have large structural variations in terms of RMSD. The contact map overlap (CMO) and the newly introduced GANGSTA score [eq. (10)] can identify structures with different SSE connectivity not detectable by methods maintaining SSE connectivity. Structure-alignment methods considering the geometry of loops that connect the regularly structured SSEs (α-helices and β-strands) in a protein have a strong bias for sequential SSE connectivity. Hence, these methods have difficulty finding structural alignments that are non-sequential in SSE connectivity.

Even if a protein fold cannot be aligned to another protein structure while maintaining the SSE connectivity, structural similarity may still exist for different SSE connectivities despite large RMSD. GANGSTA tends to align large fold motifs regardless of SSE connectivity. This is due to the following features. (1) GANGSTA does not optimize distances between residue pairs, but maximizes the number of residue pair contacts. (2) The number of gaps (i.e. the number of not aligned SSEs in the source structure) is restricted to make sure that a maximum number of of SSEs and consequently also of residues are aligned. (3) GANGSTA ignores loop structures, which helps to find structure alignments that are non-sequential in SSE connectivity.

## Methods

### General scope of method

For the protein-structure alignment problem, we call the smaller of the two protein structures the *source *structure and the larger the *target *structure. To increase flexibility of structure alignment we allow, in analogy with sequence alignment, gaps in the source structure. These gaps are assigned a penalty to ensure a global alignment. Thus, not all SSEs of the source structure are explicitly aligned. Gaps in the *target *structure occur naturally and are not subject to a penalty, since at most the number of SSEs in the *source *structure can be aligned. Note that no gaps are allowed within SSEs. In the present approach, protein structures are modeled graph-theoretically as *contact maps *on two hierarchical abstraction levels. On the residue level, the structure of a polypeptide chain with *N *residues can be represented by an (*N *× *N*) – matrix *C *of residue-pair contacts, where *C*_*ij *_is 1 if there is a contact between i and j and 0 otherwise.

Residue-pair contacts can be defined in different ways. One definition is based on the shortest distance between any atom pair of residues *i *and *j *(all atom contact), which are in contact if this distance is smaller than a given threshold *R *[57,58]. Alternatively, a contact can be defined by C_α_- or C_β_-atom pair distances of the corresponding residues [59]. In our application, a contact is established, if the C_α_-atoms of two residues are separated by less than 11 Å, a value optimized empirically for protein-structure recognition by Bastolla et al. [60]. On the secondary-structure level, a contact between two SSEs is established if there exists at least one contact on the residue level of these SSEs.

The GANGSTA procedure for protein-structure alignment is organized in two hierarchical levels. On the first level, SSEs are aligned by a GA that optimizes SSE contact similarity, yielding a selection of promising structure alignments. On the second level, equivalent SSEs are shifted relative to each other to maximize residue contact overlap.

### Graph representation of SSEs

**Figure 9:**
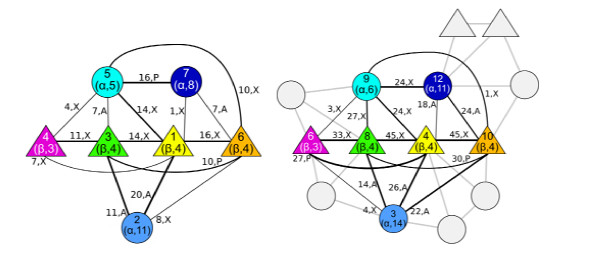
**Protein graphs**. TOPS-like graphs (Gilbert et al., 2001) of 2uagA1 (left) and 1gkuB1 (right: only aligned SSEs are labeled). The same color code as in Fig. 1 is used. Circles denote a-helices, triangles b-strands. Digits in brackets yield number of residues in SSE. Connecting lines denote edges of SSEs that are in contact. Letters on edges refer to arrangement of SSE pairs. Numbers on edges are the sum of residue pair contacts. Only edges with residue contacts are shown.

The three-dimensional arrangement of SSEs in a protein can be modeled suitably as an attributed, undirected graph G=(V,E,fTV,fLV,fCE,fOE)
 MathType@MTEF@5@5@+=feaafiart1ev1aaatCvAUfKttLearuWrP9MDH5MBPbIqV92AaeXatLxBI9gBaebbnrfifHhDYfgasaacH8akY=wiFfYdH8Gipec8Eeeu0xXdbba9frFj0=OqFfea0dXdd9vqai=hGuQ8kuc9pgc9s8qqaq=dirpe0xb9q8qiLsFr0=vr0=vr0dc8meaabaqaciaacaGaaeqabaqabeGadaaakeaacqqGhbWrcqGH9aqpcqGGOaakcqqGwbGvcqGGSaalcqqGfbqrcqGGSaalcqqGMbGzdaqhaaWcbaGaeeivaqfabaGaeeOvayfaaOGaeiilaWIaeeOzay2aa0baaSqaaiabbYeambqaaiabbAfawbaakiabcYcaSiabbAgaMnaaDaaaleaacqqGdbWqaeaacqqGfbqraaGccqGGSaalcqqGMbGzdaqhaaWcbaGaee4ta8eabaGaeeyraueaaOGaeiykaKcaaa@464D@ consisting of sets of vertices *V *and edges *E *that correspond to SSEs and contacts between SSE pairs, respectively and four attributes (maps: f). The vertices can be organized in a vector V→=(v1,v2,...vNv)
 MathType@MTEF@5@5@+=feaafiart1ev1aaatCvAUfKttLearuWrP9MDH5MBPbIqV92AaeXatLxBI9gBaebbnrfifHhDYfgasaacH8akY=wiFfYdH8Gipec8Eeeu0xXdbba9frFj0=OqFfea0dXdd9vqai=hGuQ8kuc9pgc9s8qqaq=dirpe0xb9q8qiLsFr0=vr0=vr0dc8meaabaqaciaacaGaaeqabaqabeGadaaakeaacuqGwbGvgaWcaiabg2da9iabcIcaOiabbAha2naaBaaaleaacqaIXaqmaeqaaOGaeiilaWIaeeODay3aaSbaaSqaaiabikdaYaqabaGccqGGSaalcqGGUaGlcqGGUaGlcqGGUaGlcqqG2bGDdaWgaaWcbaGaeeOta40aaSbaaWqaaiabbAha2bqabaaaleqaaOGaeiykaKcaaa@3EC0@, where component *v*_*j *_represents vertex (SSE) *j *of a given protein with *N*_*V *_SSEs numbered from N- to C-terminus. The SSEs can be defined according to DSSP [61], Stride [51] or by information in the PDB structure file. Vertices are labeled by two distinct attributes: fTV
 MathType@MTEF@5@5@+=feaafiart1ev1aaatCvAUfKttLearuWrP9MDH5MBPbIqV92AaeXatLxBI9gBaebbnrfifHhDYfgasaacH8akY=wiFfYdH8Gipec8Eeeu0xXdbba9frFj0=OqFfea0dXdd9vqai=hGuQ8kuc9pgc9s8qqaq=dirpe0xb9q8qiLsFr0=vr0=vr0dc8meaabaqaciaacaGaaeqabaqabeGadaaakeaacqqGMbGzdaqhaaWcbaGaeeivaqfabaGaeeOvayfaaaaa@308E@: V → {α, β} assigns a secondary structure type (α: α-helix, β: β-strand) and fLV
 MathType@MTEF@5@5@+=feaafiart1ev1aaatCvAUfKttLearuWrP9MDH5MBPbIqV92AaeXatLxBI9gBaebbnrfifHhDYfgasaacH8akY=wiFfYdH8Gipec8Eeeu0xXdbba9frFj0=OqFfea0dXdd9vqai=hGuQ8kuc9pgc9s8qqaq=dirpe0xb9q8qiLsFr0=vr0=vr0dc8meaabaqaciaacaGaaeqabaqabeGadaaakeaacqqGMbGzdaqhaaWcbaGaeeitaWeabaGaeeOvayfaaaaa@307E@: V → ℕ^+ ^assigns to each vertex the SSE length in terms of residue count. The vertices are connected by edges that represent contacts between SSEs. SSE contacts are defined through contacts between any pair of residues that belong to different SSEs. Likewise, edges are labeled by two attributes: fCE:E→ℕ0+
 MathType@MTEF@5@5@+=feaafiart1ev1aaatCvAUfKttLearuWrP9MDH5MBPbIqV92AaeXatLxBI9gBaebbnrfifHhDYfgasaacH8akY=wiFfYdH8Gipec8Eeeu0xXdbba9frFj0=OqFfea0dXdd9vqai=hGuQ8kuc9pgc9s8qqaq=dirpe0xb9q8qiLsFr0=vr0=vr0dc8meaabaqaciaacaGaaeqabaqabeGadaaakeaacqqGMbGzdaqhaaWcbaGaee4qameabaGaeeyraueaaOGaeiOoaOJaeeyrauKaeyOKH46efv3ySLgznfgDOjdaryqr1ngBPrginfgDObcv39gaiqaacqWFveItdaqhaaWcbaGaeGimaadabaGaey4kaScaaaaa@40F6@ assigns the number of residue pair contacts to the SSE pair, while f0E
 MathType@MTEF@5@5@+=feaafiart1ev1aaatCvAUfKttLearuWrP9MDH5MBPbIqV92AaeXatLxBI9gBaebbnrfifHhDYfgasaacH8akY=wiFfYdH8Gipec8Eeeu0xXdbba9frFj0=OqFfea0dXdd9vqai=hGuQ8kuc9pgc9s8qqaq=dirpe0xb9q8qiLsFr0=vr0=vr0dc8meaabaqaciaacaGaaeqabaqabeGadaaakeaacqqGMbGzdaqhaaWcbaGaeGimaadabaGaeeyraueaaaaa@302B@: E → {0, X, A, P} assigns the relative orientation (conformation) between two SSEs [26]. The following three conformations are distinguished: anti-parallel (*A*), parallel (*P*), neither parallel nor antiparallel (crossed, *X*) while *0 *marks edges (SSE pairs) that have no residue contacts. Fig. 9 shows the protein graphs for 2uagA1 and 1gkuB1.

### Structure alignment on SSE level (1^st ^level of the hierarchy)

The problem of finding a structure alignment for a source protein structure (*s*) onto a target protein structure (*t*) of equal or larger size, represented by the graphs *G*^s ^and *G*^t^, can be understood as the task to find the maximum common subgraph (maximum subset of *V*^s ^and *V*^t^) *G*^st ^⊆ *G*^s^, *G*^t^. Thus, a structure alignment can be specified as subgraph isomorphism *g*^st^: *G*^s ^→ *G*^t ^composed of two maps: *g*_*V*_: *V*^s ^→ *V*^t ^and *g*_*E*_: *E*^s ^→ *E*^t ^relating structural details between the two considered proteins. There are two constraining conditions that must be fulfilled for a valid structure alignment: only the same type of SSEs (α or β) can be matched

fTV(v)=fTV(gV(v))
 MathType@MTEF@5@5@+=feaafiart1ev1aaatCvAUfKttLearuWrP9MDH5MBPbIqV92AaeXatLxBI9gBaebbnrfifHhDYfgasaacH8akY=wiFfYdH8Gipec8Eeeu0xXdbba9frFj0=OqFfea0dXdd9vqai=hGuQ8kuc9pgc9s8qqaq=dirpe0xb9q8qiLsFr0=vr0=vr0dc8meaabaqaciaacaGaaeqabaqabeGadaaakeaacqqGMbGzdaqhaaWcbaGaeeivaqfabaGaeeOvayfaaOGaeiikaGIaeeODayNaeiykaKIaeyypa0JaeeOzay2aa0baaSqaaiabbsfaubqaaiabbAfawbaakiabcIcaOiabdEgaNnaaBaaaleaacqqGwbGvaeqaaOGaeiikaGIaeeODayNaeiykaKIaeiykaKcaaa@4046@ ∀ considered v ∈ V^s ^    (1)

and equivalent SSEs cannot have length differences exceeding ten residues

|fLV
 MathType@MTEF@5@5@+=feaafiart1ev1aaatCvAUfKttLearuWrP9MDH5MBPbIqV92AaeXatLxBI9gBaebbnrfifHhDYfgasaacH8akY=wiFfYdH8Gipec8Eeeu0xXdbba9frFj0=OqFfea0dXdd9vqai=hGuQ8kuc9pgc9s8qqaq=dirpe0xb9q8qiLsFr0=vr0=vr0dc8meaabaqaciaacaGaaeqabaqabeGadaaakeaacqqGMbGzdaqhaaWcbaGaeeitaWeabaGaeeOvayfaaaaa@307E@(v) - fLV
 MathType@MTEF@5@5@+=feaafiart1ev1aaatCvAUfKttLearuWrP9MDH5MBPbIqV92AaeXatLxBI9gBaebbnrfifHhDYfgasaacH8akY=wiFfYdH8Gipec8Eeeu0xXdbba9frFj0=OqFfea0dXdd9vqai=hGuQ8kuc9pgc9s8qqaq=dirpe0xb9q8qiLsFr0=vr0=vr0dc8meaabaqaciaacaGaaeqabaqabeGadaaakeaacqqGMbGzdaqhaaWcbaGaeeitaWeabaGaeeOvayfaaaaa@307E@(g_v_(v))| ≤ 10 ∀ considered v ∈ V^s ^    (2)

These two conditions must hold only for SSEs that are explicitly considered in a structure alignment. If gaps are introduced, some SSEs in the source structure are ignored. Furthermore, the difference in contacts

∑e∈Es|fCE(e)−fCE(gE(e))|     (3)
 MathType@MTEF@5@5@+=feaafiart1ev1aaatCvAUfKttLearuWrP9MDH5MBPbIqV92AaeXatLxBI9gBaebbnrfifHhDYfgasaacH8akY=wiFfYdH8Gipec8Eeeu0xXdbba9frFj0=OqFfea0dXdd9vqai=hGuQ8kuc9pgc9s8qqaq=dirpe0xb9q8qiLsFr0=vr0=vr0dc8meaabaqaciaacaGaaeqabaqabeGadaaakeaadaaeqbqaamaaemaabaGaeeOzay2aa0baaSqaaiabboeadbqaaiabbweafbaakiabcIcaOiabbwgaLjabcMcaPiabgkHiTiabbAgaMnaaDaaaleaacqqGdbWqaeaacqqGfbqraaGccqGGOaakcqqGNbWzdaWgaaWcbaGaeeyraueabeaakiabcIcaOiabbwgaLjabcMcaPiabcMcaPaGaay5bSlaawIa7aaWcbaGaeeyzauMaeyicI4Saeeyrau0aaWbaaWqabeaacqqGZbWCaaaaleqaniabggHiLdGccaWLjaGaaCzcamaabmaabaGaeG4mamdacaGLOaGaayzkaaaaaa@4DD9@

and the SSE pair orientation mismatch

∑e∈Es|fOE(e)⊙fOE(gE(e))|     (4)
 MathType@MTEF@5@5@+=feaafiart1ev1aaatCvAUfKttLearuWrP9MDH5MBPbIqV92AaeXatLxBI9gBaebbnrfifHhDYfgasaacH8akY=wiFfYdH8Gipec8Eeeu0xXdbba9frFj0=OqFfea0dXdd9vqai=hGuQ8kuc9pgc9s8qqaq=dirpe0xb9q8qiLsFr0=vr0=vr0dc8meaabaqaciaacaGaaeqabaqabeGadaaakeaadaaeqbqaamaaemaabaGaeeOzay2aa0baaSqaaiabb+eapbqaaiabbweafbaakiabcIcaOiabbwgaLjabcMcaPiablMPiLjabbAgaMnaaDaaaleaacqqGpbWtaeaacqqGfbqraaGccqGGOaakcqqGNbWzdaWgaaWcbaGaeeyraueabeaakiabcIcaOiabbwgaLjabcMcaPiabcMcaPaGaay5bSlaawIa7aaWcbaGaeeyzauMaeyicI4Saeeyrau0aaWbaaWqabeaacqqGZbWCaaaaleqaniabggHiLdGccaWLjaGaaCzcamaabmaabaGaeGinaqdacaGLOaGaayzkaaaaaa@4ECF@

has to be minimized. In eq. (4) the binary operation ⊙ compares the SSE pair orientation, *x*, *y *∈ {*O*, *X*, *A*, *P*}, of two structures

x⊙y:={1if x=y0.5if (x≠y)∧(x=X∨y=X)0else;x,y∈{0,X,A,P}     (5)
 MathType@MTEF@5@5@+=feaafiart1ev1aaatCvAUfKttLearuWrP9MDH5MBPbIqV92AaeXatLxBI9gBaebbnrfifHhDYfgasaacH8akY=wiFfYdH8Gipec8Eeeu0xXdbba9frFj0=OqFfea0dXdd9vqai=hGuQ8kuc9pgc9s8qqaq=dirpe0xb9q8qiLsFr0=vr0=vr0dc8meaabaqaciaacaGaaeqabaqabeGadaaakeaacqqG4baEcqWIzkszcqqG5bqEcqGG6aGocqGH9aqpdaGabeqaauaabaqadiaaaeaacqaIXaqmaeaacqqGPbqAcqqGMbGzcqqGGaaicqqG4baEcqGH9aqpcqqG5bqEaeaacqaIWaamcqGGUaGlcqaI1aqnaeaacqqGPbqAcqqGMbGzcqqGGaaicqGGOaakcqqG4baEcqGHGjsUcqqG5bqEcqGGPaqkcqGHNis2cqGGOaakcqqG4baEcqGH9aqpcqqGybawcqGHOiI2cqqG5bqEcqGH9aqpcqqGybawcqGGPaqkaeaacqaIWaamaeaacqqGLbqzcqqGSbaBcqqGZbWCcqqGLbqzcqGG7aWocaWLjaGaeeiEaGNaeiilaWIaeeyEaKNaeyicI4Saei4EaSNaeGimaaJaeiilaWIaeeiwaGLaeiilaWIaeeyqaeKaeiilaWIaeeiuaaLaeiyFa0haaaGaay5EaaGaaCzcaiaaxMaadaqadaqaaiabiwda1aGaayjkaiaawMcaaaaa@70FF@

To evaluate the quality of a given structure alignment for a pair of proteins (s,t), represented by the graph monomorphism *g*^st^, we use the following objective function

obj(gst,VS,ES)=wC(1−∑e∈ES|fCE(e)−fCE(gE(e))|∑e∈ESfCE(e)+∑e∈ESfCE(gE(e)))+wO(∑e∈ES|fOE(e)⊙fOE(gE(e))||{e∈ES|fOE(e)≠0}|)−L(VS,gst)−GP+Seq(VS,gst).     (6)
 MathType@MTEF@5@5@+=feaafiart1ev1aaatCvAUfKttLearuWrP9MDH5MBPbIqV92AaeXatLxBI9gBaebbnrfifHhDYfgasaacH8akY=wiFfYdH8Gipec8Eeeu0xXdbba9frFj0=OqFfea0dXdd9vqai=hGuQ8kuc9pgc9s8qqaq=dirpe0xb9q8qiLsFr0=vr0=vr0dc8meaabaqaciaacaGaaeqabaqabeGadaaakeaacqqGVbWBcqqGIbGycqqGQbGAcqGGOaakcqqGNbWzdaahaaWcbeqaaiabbohaZjabbsha0baakiabcYcaSiabbAfawnaaCaaaleqabaGaee4uamfaaOGaeiilaWIaeeyrau0aaWbaaSqabeaacqqGtbWuaaGccqGGPaqkcqGH9aqpcqqG3bWDdaWgaaWcbaGaee4qameabeaakmaabmaabaGaeGymaeJaeyOeI0YaaSaaaeaadaaeqbqaamaaemaabaGaeeOzay2aa0baaSqaaiabboeadbqaaiabbweafbaakiabcIcaOiabbwgaLjabcMcaPiabgkHiTiabbAgaMnaaDaaaleaacqqGdbWqaeaacqqGfbqraaGccqGGOaakcqqGNbWzdaWgaaWcbaGaeeyraueabeaakiabcIcaOiabbwgaLjabcMcaPiabcMcaPaGaay5bSlaawIa7aaWcbaGaeeyzauMaeyicI4Saeeyrau0aaWbaaWqabeaacqqGtbWuaaaaleqaniabggHiLdaakeaadaaeqbqaaiabbAgaMnaaDaaaleaacqqGdbWqaeaacqqGfbqraaGccqGGOaakcqqGLbqzcqGGPaqkaSqaaiabbwgaLjabgIGiolabbweafnaaCaaameqabaGaee4uamfaaaWcbeqdcqGHris5aOGaey4kaSYaaabuaeaacqqGMbGzdaqhaaWcbaGaee4qameabaGaeeyraueaaOGaeiikaGIaee4zaC2aaSbaaSqaaiabbweafbqabaGccqGGOaakcqqGLbqzcqGGPaqkcqGGPaqkaSqaaiabbwgaLjabgIGiolabbweafnaaCaaameqabaGaee4uamfaaaWcbeqdcqGHris5aaaaaOGaayjkaiaawMcaaiabgUcaRiabbEha3naaBaaaleaacqqGpbWtaeqaaOWaaeWaaeaadaWcaaqaamaaqafabaWaaqWaaeaacqqGMbGzdaqhaaWcbaGaee4ta8eabaGaeeyraueaaOGaeiikaGIaeeyzauMaeiykaKIaeSyMIuMaeeOzay2aa0baaSqaaiabb+eapbqaaiabbweafbaakiabcIcaOiabbEgaNnaaBaaaleaacqqGfbqraeqaaOGaeiikaGIaeeyzauMaeiykaKIaeiykaKcacaGLhWUaayjcSdaaleaacqqGLbqzcqGHiiIZcqqGfbqrdaahaaadbeqaaiabbofatbaaaSqab0GaeyyeIuoaaOqaamaaemaabaWaaiWabeaacqqGLbqzcqGHiiIZcqqGfbqrdaahaaWcbeqaaiabbofatbaakmaaeeaabaGaeeOzay2aa0baaSqaaiabb+eapbqaaiabbweafbaakiabcIcaOiabbwgaLjabcMcaPiabgcMi5kabicdaWaGaay5bSdaacaGL7bGaayzFaaaacaGLhWUaayjcSdaaaaGaayjkaiaawMcaaiabgkHiTiabbYeamjabcIcaOiabbAfawnaaCaaaleqabaGaee4uamfaaOGaeiilaWIaee4zaC2aaWbaaSqabeaacqqGZbWCcqqG0baDaaGccqGGPaqkcqGHsislcqqGhbWrcqqGqbaucqGHRaWkcqqGtbWucqqGLbqzcqqGXbqCcqGGOaakcqqGwbGvdaahaaWcbeqaaiabbofatbaakiabcYcaSiabbEgaNnaaCaaaleqabaGaee4CamNaeeiDaqhaaOGaeiykaKIaeiOla4IaaCzcaiaaxMaadaqadaqaaiabiAda2aGaayjkaiaawMcaaaaa@DE55@

The first term in the objective function measures the structural similarity between source and target proteins by comparing the number of contacts between aligned SSEs. It is normalized to yield unity for contact identity (each contact in the source structure can be mapped on the target structure) and zero for no common contacts. The second term considers similarity in the relative orientation of SSE pairs in source and target structures, again normalized to yield unity for a perfect match and zero, if none of the orientations agree. These two terms are tuned by the weights *w*_*C *_and *w*_*O*_. Matching SSEs with length differences above a threshold is penalized depending on SSE type by the parameter *L*. A small number of SSEs from the source structure may not be considered for structure alignment. Those gaps are penalized by the gap penalty factor *GP *to ensure that the GA tries to find the maximum common subgraph instead of an arbitrary, small subgraph. Depending on its sign, the term *Seq *is a weight to favor sequential or non-sequential solutions [see Additional file 1, implementation details]. The parameters *w*_*C*_, *w*_*O*_, and penalty factors *L*, *GP*, *Seq *in eq. (6) were optimized to yield maximum separation of structure pairs belonging to the same SCOP superfamily from those belonging to different SCOP superfamilies (see Fig. 2) referring to the GANGSTA score, eq. (10).

### Genetic algorithm

GAs are heuristic methods to tackle difficult optimization problems. GAs use principles of evolution to create a set of individuals and to let them evolve from generation to generation using specific gene operations. Individuals are possible solutions (generally sub-optimal) of the optimization problem. In our case individuals can be identified with a specific graph monomorphism *g*^st ^probing the similarity between two protein structures (*s*ource and *t*arget), which can be represented in terms of a vector

g→st=(g1...g|VS|)
 MathType@MTEF@5@5@+=feaafiart1ev1aaatCvAUfKttLearuWrP9MDH5MBPbIqV92AaeXatLxBI9gBaebbnrfifHhDYfgasaacH8akY=wiFfYdH8Gipec8Eeeu0xXdbba9frFj0=OqFfea0dXdd9vqai=hGuQ8kuc9pgc9s8qqaq=dirpe0xb9q8qiLsFr0=vr0=vr0dc8meaabaqaciaacaGaaeqabaqabeGadaaakeaacuqGNbWzgaWcamaaCaaaleqabaGaee4CamNaeeiDaqhaaOGaeyypa0JaeiikaGIaee4zaC2aaSbaaSqaaiabigdaXaqabaGccqGGUaGlcqGGUaGlcqGGUaGlcqqGNbWzdaWgaaWcbaGaeiiFaWNaeeOvay1aaWbaaWqabeaacqqGtbWuaaWccqGG8baFaeqaaOGaeiykaKcaaa@4029@, where *g*_*j *_∈ ℕ.     (7)

The |*V*^*s*^| components of g→st
 MathType@MTEF@5@5@+=feaafiart1ev1aaatCvAUfKttLearuWrP9MDH5MBPbIqV92AaeXatLxBI9gBaebbnrfifHhDYfgasaacH8akY=wiFfYdH8Gipec8Eeeu0xXdbba9frFj0=OqFfea0dXdd9vqai=hGuQ8kuc9pgc9s8qqaq=dirpe0xb9q8qiLsFr0=vr0=vr0dc8meaabaqaciaacaGaaeqabaqabeGadaaakeaacuqGNbWzgaWcamaaCaaaleqabaGaee4CamNaeeiDaqhaaaaa@311C@ refer to the |*V*^*s*^| vertices (SSEs) of the source protein. The integer *g*_*j *_assigns SSE *g*_*j *_from the target protein (*t*) to SSE j from the source protein (*s*). Both SSEs have to fulfill relation (1) and (2), i.e. to be of the same type and of similar length. The assignment of a specific SSE from a source structure to an SSE of a target structure, denoted by components of g→st
 MathType@MTEF@5@5@+=feaafiart1ev1aaatCvAUfKttLearuWrP9MDH5MBPbIqV92AaeXatLxBI9gBaebbnrfifHhDYfgasaacH8akY=wiFfYdH8Gipec8Eeeu0xXdbba9frFj0=OqFfea0dXdd9vqai=hGuQ8kuc9pgc9s8qqaq=dirpe0xb9q8qiLsFr0=vr0=vr0dc8meaabaqaciaacaGaaeqabaqabeGadaaakeaacuqGNbWzgaWcamaaCaaaleqabaGaee4CamNaeeiDaqhaaaaa@311C@, can be considered as a gene. The possible values of a gene gjst
 MathType@MTEF@5@5@+=feaafiart1ev1aaatCvAUfKttLearuWrP9MDH5MBPbIqV92AaeXatLxBI9gBaebbnrfifHhDYfgasaacH8akY=wiFfYdH8Gipec8Eeeu0xXdbba9frFj0=OqFfea0dXdd9vqai=hGuQ8kuc9pgc9s8qqaq=dirpe0xb9q8qiLsFr0=vr0=vr0dc8meaabaqaciaacaGaaeqabaqabeGadaaakeaacqqGNbWzdaqhaaWcbaGaeeOAaOgabaGaee4CamNaeeiDaqhaaaaa@3265@, the alleles, for a pair of source and target proteins (*s*, *t*) are the set of integers

allelesjst={k|[vjs∈Vsrc,vkt∈Vtarg]∧[fTV(vjs)=fTV(vkt)]∧[|fLV(vjs)−fLV(vkt)|≤10]}     (8)
 MathType@MTEF@5@5@+=feaafiart1ev1aaatCvAUfKttLearuWrP9MDH5MBPbIqV92AaeXatLxBI9gBaebbnrfifHhDYfgasaacH8akY=wiFfYdH8Gipec8Eeeu0xXdbba9frFj0=OqFfea0dXdd9vqai=hGuQ8kuc9pgc9s8qqaq=dirpe0xb9q8qiLsFr0=vr0=vr0dc8meaabaqaciaacaGaaeqabaqabeGadaaakeaacqqGHbqycqqGSbaBcqqGSbaBcqqGLbqzcqqGSbaBcqqGLbqzcqqGZbWCdaqhaaWcbaGaeeOAaOgabaGaee4CamNaeeiDaqhaaOGaeyypa0ZaaiWabeaafaqabeGabaaabaGaee4AaS2aaqqaaeaadaWadaqaaiabbAha2naaDaaaleaacqqGQbGAaeaacqqGZbWCaaGccqGHiiIZcqqGwbGvdaahaaWcbeqaaiabbohaZjabbkhaYjabbogaJbaakiabcYcaSiabbAha2naaDaaaleaacqqGRbWAaeaacqqG0baDaaGccqGHiiIZcqqGwbGvdaahaaWcbeqaaiabbsha0jabbggaHjabbkhaYjabbEgaNbaaaOGaay5waiaaw2faaaGaay5bSdGaey4jIK9aamWaaeaacqqGMbGzdaqhaaWcbaGaeeivaqfabaGaeeOvayfaaOGaeiikaGIaeeODay3aa0baaSqaaiabbQgaQbqaaiabbohaZbaakiabcMcaPiabg2da9iabbAgaMnaaDaaaleaacqqGubavaeaacqqGwbGvaaGccqGGOaakcqqG2bGDdaqhaaWcbaGaee4AaSgabaGaeeiDaqhaaOGaeiykaKcacaGLBbGaayzxaaaabaGaey4jIK9aamWaaeaacqGG8baFcqqGMbGzdaqhaaWcbaGaeeitaWeabaGaeeOvayfaaOGaeiikaGIaeeODay3aa0baaSqaaiabbQgaQbqaaiabbohaZbaakiabcMcaPiabgkHiTiabbAgaMnaaDaaaleaacqqGmbataeaacqqGwbGvaaGccqGGOaakcqqG2bGDdaqhaaWcbaGaee4AaSgabaGaeeiDaqhaaOGaeiykaKIaeiiFaWNaeyizImQaeGymaeJaeGimaadacaGLBbGaayzxaaaaaaGaay5Eaiaaw2haaiaaxMaacaWLjaWaaeWaaeaacqaI4aaoaiaawIcacaGLPaaaaaa@97B1@

A new generation evolves by gene exchange and mutations applied to individuals to find improved solutions with larger values of the objective function, eq. (6). The newly generated children and the fittest parents form the next generation. This procedure is repeated until the optimum is found or a suitable stop criterion is reached. We use the following gene operators in our GA:

1 Gene operators exchanging genes between pairs of individuals:

1A Random crossover: A random number of randomly selected components of g→st
 MathType@MTEF@5@5@+=feaafiart1ev1aaatCvAUfKttLearuWrP9MDH5MBPbIqV92AaeXatLxBI9gBaebbnrfifHhDYfgasaacH8akY=wiFfYdH8Gipec8Eeeu0xXdbba9frFj0=OqFfea0dXdd9vqai=hGuQ8kuc9pgc9s8qqaq=dirpe0xb9q8qiLsFr0=vr0=vr0dc8meaabaqaciaacaGaaeqabaqabeGadaaakeaacuqGNbWzgaWcamaaCaaaleqabaGaee4CamNaeeiDaqhaaaaa@311C@ are exchanged.

1B Two-point crossover: Two components of g→st
 MathType@MTEF@5@5@+=feaafiart1ev1aaatCvAUfKttLearuWrP9MDH5MBPbIqV92AaeXatLxBI9gBaebbnrfifHhDYfgasaacH8akY=wiFfYdH8Gipec8Eeeu0xXdbba9frFj0=OqFfea0dXdd9vqai=hGuQ8kuc9pgc9s8qqaq=dirpe0xb9q8qiLsFr0=vr0=vr0dc8meaabaqaciaacaGaaeqabaqabeGadaaakeaacuqGNbWzgaWcamaaCaaaleqabaGaee4CamNaeeiDaqhaaaaa@311C@ are randomly selected. All components between those two form the "crossover region" and are exchanged.

1C Helix crossover: All components of g→st
 MathType@MTEF@5@5@+=feaafiart1ev1aaatCvAUfKttLearuWrP9MDH5MBPbIqV92AaeXatLxBI9gBaebbnrfifHhDYfgasaacH8akY=wiFfYdH8Gipec8Eeeu0xXdbba9frFj0=OqFfea0dXdd9vqai=hGuQ8kuc9pgc9s8qqaq=dirpe0xb9q8qiLsFr0=vr0=vr0dc8meaabaqaciaacaGaaeqabaqabeGadaaakeaacuqGNbWzgaWcamaaCaaaleqabaGaee4CamNaeeiDaqhaaaaa@311C@ of helix type are exchanged.

2 Gene mutation operators applied to a single individual:

2A Random mutation: A small, random number of g→st
 MathType@MTEF@5@5@+=feaafiart1ev1aaatCvAUfKttLearuWrP9MDH5MBPbIqV92AaeXatLxBI9gBaebbnrfifHhDYfgasaacH8akY=wiFfYdH8Gipec8Eeeu0xXdbba9frFj0=OqFfea0dXdd9vqai=hGuQ8kuc9pgc9s8qqaq=dirpe0xb9q8qiLsFr0=vr0=vr0dc8meaabaqaciaacaGaaeqabaqabeGadaaakeaacuqGNbWzgaWcamaaCaaaleqabaGaee4CamNaeeiDaqhaaaaa@311C@ components are set to randomly selected alleles, eq. (8).

2B Exchange mutation: Two components of g→st
 MathType@MTEF@5@5@+=feaafiart1ev1aaatCvAUfKttLearuWrP9MDH5MBPbIqV92AaeXatLxBI9gBaebbnrfifHhDYfgasaacH8akY=wiFfYdH8Gipec8Eeeu0xXdbba9frFj0=OqFfea0dXdd9vqai=hGuQ8kuc9pgc9s8qqaq=dirpe0xb9q8qiLsFr0=vr0=vr0dc8meaabaqaciaacaGaaeqabaqabeGadaaakeaacuqGNbWzgaWcamaaCaaaleqabaGaee4CamNaeeiDaqhaaaaa@311C@ that are type and length compatible are exchanged.

2C Greedy mutation: For a random component *g*_*j *_(gene) the allele with the highest value of the objective function obj(g→
 MathType@MTEF@5@5@+=feaafiart1ev1aaatCvAUfKttLearuWrP9MDH5MBPbIqV92AaeXatLxBI9gBaebbnrfifHhDYfgasaacH8akY=wiFfYdH8Gipec8Eeeu0xXdbba9frFj0=OqFfea0dXdd9vqai=hGuQ8kuc9pgc9s8qqaq=dirpe0xb9q8qiLsFr0=vr0=vr0dc8meaabaqaciaacaGaaeqabaqabeGadaaakeaacuWGNbWzgaWcaaaa@2E15@) is selected. If this allele is already in use for another gene, an exchange of the two genes is performed.

Some of the gene operators create children that do not agree with our constraints (for instance a duplicate usage of one SSE in the same structure violating the injectivity of the monomorphism). Those "lethal" children are discarded.

### Structure alignment on residue level (2^nd ^level of the hierarchy)

The result from the GA is a structure alignment on the SSE level. Often there are length differences among pairs of matched SSEs. In this case, the shorter SSE is shifted along the longer SSE to find an optimal arrangement with respect to residue pair contacts. Two methods are used to solve the problem. All possible combinations of residue assignments for each pair of SSEs from the structure alignment are considered to find the most similar residue pair contact map.

Gaps in an individual SSE on the residue level would result in an SSE consisting rather of two instead of one SSE (if the gap is close to the center of the SSE) or in an effectively shorter SSE (if the gap is introduced on the edge of the SSE). These situations are considered on the SSE level as two independent SSEs or as a shorter SSE, respectively. Hence, no gaps in SSEs need to be considered.

**Figure 10:**
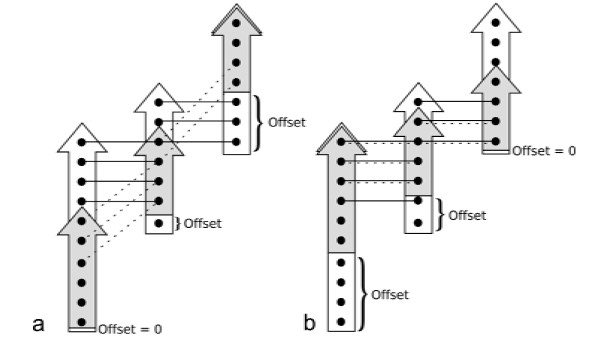
**Possible residue shifts for an aligned pair of SSEs from two different protein structures**. Two proteins (white: source protein, gray: target protein) consisting of three SSEs each. Each bold dot within a SSE represents a residue. Lines connect residue pairs of different SSEs that are in contact. Solid lines refer to contacts in the source protein; dotted lines refer to contacts in the target protein. For conserved contacts, residue pairs are connected by horizontal solid and dotted lines, simultaneously. Part a: No contacts of source and target proteins are conserved (qresst
 MathType@MTEF@5@5@+=feaafiart1ev1aaatCvAUfKttLearuWrP9MDH5MBPbIqV92AaeXatLxBI9gBaebbnrfifHhDYfgasaacH8akY=wiFfYdH8Gipec8Eeeu0xXdbba9frFj0=OqFfea0dXdd9vqai=hGuQ8kuc9pgc9s8qqaq=dirpe0xb9q8qiLsFr0=vr0=vr0dc8meaabaqaciaacaGaaeqabaqabeGadaaakeaacqqGXbqCdaqhaaWcbaGaeeOCaiNaeeyzauMaee4CamhabaGaee4CamNaeeiDaqhaaaaa@3547@ = 0). Part b: A maximum of five contacts from a total number of seven contacts are conserved (qresst
 MathType@MTEF@5@5@+=feaafiart1ev1aaatCvAUfKttLearuWrP9MDH5MBPbIqV92AaeXatLxBI9gBaebbnrfifHhDYfgasaacH8akY=wiFfYdH8Gipec8Eeeu0xXdbba9frFj0=OqFfea0dXdd9vqai=hGuQ8kuc9pgc9s8qqaq=dirpe0xb9q8qiLsFr0=vr0=vr0dc8meaabaqaciaacaGaaeqabaqabeGadaaakeaacqqGXbqCdaqhaaWcbaGaeeOCaiNaeeyzauMaee4CamhabaGaee4CamNaeeiDaqhaaaaa@3547@ = 5/7).

The residue contact map overlap qresst
 MathType@MTEF@5@5@+=feaafiart1ev1aaatCvAUfKttLearuWrP9MDH5MBPbIqV92AaeXatLxBI9gBaebbnrfifHhDYfgasaacH8akY=wiFfYdH8Gipec8Eeeu0xXdbba9frFj0=OqFfea0dXdd9vqai=hGuQ8kuc9pgc9s8qqaq=dirpe0xb9q8qiLsFr0=vr0=vr0dc8meaabaqaciaacaGaaeqabaqabeGadaaakeaacqqGXbqCdaqhaaWcbaGaeeOCaiNaeeyzauMaee4CamhabaGaee4CamNaeeiDaqhaaaaa@3547@, which is a measure for the residue pair contacts that are conserved in a structure alignment, is defined by [60].

qresst=∑i,jCi,jsCmap(i),map(j)tmax⁡[∑i,jCi,js,∑i,jCmap(i),map(j)t],     (9)
 MathType@MTEF@5@5@+=feaafiart1ev1aaatCvAUfKttLearuWrP9MDH5MBPbIqV92AaeXatLxBI9gBaebbnrfifHhDYfgasaacH8akY=wiFfYdH8Gipec8Eeeu0xXdbba9frFj0=OqFfea0dXdd9vqai=hGuQ8kuc9pgc9s8qqaq=dirpe0xb9q8qiLsFr0=vr0=vr0dc8meaabaqaciaacaGaaeqabaqabeGadaaakeaacqqGXbqCdaqhaaWcbaGaeeOCaiNaeeyzauMaee4CamhabaGaee4CamNaeeiDaqhaaOGaeyypa0ZaaSaaaeaadaaeqbqaaiabboeadnaaDaaaleaacqqGPbqAcqGGSaalcqqGQbGAaeaacqqGZbWCaaGccqqGdbWqdaqhaaWcbaGaeeyBa0MaeeyyaeMaeeiCaaNaeiikaGIaeeyAaKMaeiykaKIaeiilaWIaeeyBa0MaeeyyaeMaeeiCaaNaeiikaGIaeeOAaOMaeiykaKcabaGaeeiDaqhaaaqaaiabbMgaPjabcYcaSiabbQgaQbqab0GaeyyeIuoaaOqaaiGbc2gaTjabcggaHjabcIha4naadmaabaWaaabuaeaacqqGdbWqdaqhaaWcbaGaeeyAaKMaeiilaWIaeeOAaOgabaGaee4CamhaaaqaaiabbMgaPjabcYcaSiabbQgaQbqab0GaeyyeIuoakiabcYcaSmaaqafabaGaee4qam0aa0baaSqaaiabb2gaTjabbggaHjabbchaWjabcIcaOiabbMgaPjabcMcaPiabcYcaSiabb2gaTjabbggaHjabbchaWjabcIcaOiabbQgaQjabcMcaPaqaaiabbsha0baaaeaacqqGPbqAcqGGSaalcqqGQbGAaeqaniabggHiLdaakiaawUfacaGLDbaaaaGaeiilaWIaaCzcaiaaxMaadaqadaqaaiabiMda5aGaayjkaiaawMcaaaaa@8334@

where *C*^*s *^and *C*^*t *^are C_α _contact maps of the source and target protein structures, respectively. The combination of SSE assignment (g→st
 MathType@MTEF@5@5@+=feaafiart1ev1aaatCvAUfKttLearuWrP9MDH5MBPbIqV92AaeXatLxBI9gBaebbnrfifHhDYfgasaacH8akY=wiFfYdH8Gipec8Eeeu0xXdbba9frFj0=OqFfea0dXdd9vqai=hGuQ8kuc9pgc9s8qqaq=dirpe0xb9q8qiLsFr0=vr0=vr0dc8meaabaqaciaacaGaaeqabaqabeGadaaakeaacuqGNbWzgaWcamaaCaaaleqabaGaee4CamNaeeiDaqhaaaaa@311C@) and shifts of matched SSEs results in a map, which assigns residue *j *of the source protein to residue *map*(*j*) of the target protein. The objective of the second level of hierarchy is to maximize the residue contact overlap qresst
 MathType@MTEF@5@5@+=feaafiart1ev1aaatCvAUfKttLearuWrP9MDH5MBPbIqV92AaeXatLxBI9gBaebbnrfifHhDYfgasaacH8akY=wiFfYdH8Gipec8Eeeu0xXdbba9frFj0=OqFfea0dXdd9vqai=hGuQ8kuc9pgc9s8qqaq=dirpe0xb9q8qiLsFr0=vr0=vr0dc8meaabaqaciaacaGaaeqabaqabeGadaaakeaacqqGXbqCdaqhaaWcbaGaeeOCaiNaeeyzauMaee4CamhabaGaee4CamNaeeiDaqhaaaaa@3547@, eq. (9). In Fig. 10 two examples for the same SSE mapping are shown with different SSE shifts.

### GANGSTA score

The last step in the structure alignment procedure is a superposition of source and target protein structures minimizing the RMSD of the C_α _atoms (*RMSD*(*C*_α_)) with the Kabsch algorithm [28]. To rank the quality of multiple pairwise structure alignments the value of the objective function, eq. (6), is a crude method working on the SSE level, designed for fast screening of many individuals occurring in the GA. The residue contact map overlap qresst
 MathType@MTEF@5@5@+=feaafiart1ev1aaatCvAUfKttLearuWrP9MDH5MBPbIqV92AaeXatLxBI9gBaebbnrfifHhDYfgasaacH8akY=wiFfYdH8Gipec8Eeeu0xXdbba9frFj0=OqFfea0dXdd9vqai=hGuQ8kuc9pgc9s8qqaq=dirpe0xb9q8qiLsFr0=vr0=vr0dc8meaabaqaciaacaGaaeqabaqabeGadaaakeaacqqGXbqCdaqhaaWcbaGaeeOCaiNaeeyzauMaee4CamhabaGaee4CamNaeeiDaqhaaaaa@3547@ works on the residue level, but focuses on short distances only. In absence of chain connectivity, as is the case for structure alignment of SSEs, a short distance criterion alone is not sufficiently accurate to characterize global topologies of protein structures. Therefore, we have introduced a more detailed measure of protein-structure similarity that considers simultaneously RMSD(C_α_) [Å], number of not aligned SSEs in the source protein *N*_*gap*_, residue contact map overlap qresst
 MathType@MTEF@5@5@+=feaafiart1ev1aaatCvAUfKttLearuWrP9MDH5MBPbIqV92AaeXatLxBI9gBaebbnrfifHhDYfgasaacH8akY=wiFfYdH8Gipec8Eeeu0xXdbba9frFj0=OqFfea0dXdd9vqai=hGuQ8kuc9pgc9s8qqaq=dirpe0xb9q8qiLsFr0=vr0=vr0dc8meaabaqaciaacaGaaeqabaqabeGadaaakeaacqqGXbqCdaqhaaWcbaGaeeOCaiNaeeyzauMaee4CamhabaGaee4CamNaeeiDaqhaaaaa@3547@ and relative difference in SSE pair distances Δ*SSE *between source and target structure

Score=RMSD(Cα)+2∗NgapNalnRes∗qresst∗(1−ΔSSE)+ε.     (10)
 MathType@MTEF@5@5@+=feaafiart1ev1aaatCvAUfKttLearuWrP9MDH5MBPbIqV92AaeXatLxBI9gBaebbnrfifHhDYfgasaacH8akY=wiFfYdH8Gipec8Eeeu0xXdbba9frFj0=OqFfea0dXdd9vqai=hGuQ8kuc9pgc9s8qqaq=dirpe0xb9q8qiLsFr0=vr0=vr0dc8meaabaqaciaacaGaaeqabaqabeGadaaakeaacqqGtbWucqqGJbWycqqGVbWBcqqGYbGCcqqGLbqzcqGH9aqpdaWcaaqaaiabbkfasjabb2eanjabbofatjabbseaejabcIcaOiabboeadnaaBaaaleaacqaHXoqyaeqaaOGaeiykaKIaey4kaSIaeGOmaiJaey4fIOIaeeOta40aaSbaaSqaaiabbEgaNjabbggaHjabbchaWbqabaaakeaacqqGobGtdaWgaaWcbaGaeeyyaeMaeeiBaWMaeeOBa4MaeeOuaiLaeeyzauMaee4CamhabeaakiabgEHiQiabbghaXnaaDaaaleaacqqGYbGCcqqGLbqzcqqGZbWCaeaacqqGZbWCcqqG0baDaaGccqGHxiIkcqGGOaakcqaIXaqmcqGHsislcqqHuoarcqqGtbWucqqGtbWucqqGfbqrcqGGPaqkcqGHRaWkiiaacqWF1oqzaaGaeiOla4IaaCzcaiaaxMaadaqadaqaaiabigdaXiabicdaWaGaayjkaiaawMcaaaaa@69E7@

This GANGSTA score is normalized by the number of aligned residues *N*_*alnRes *_and a small ε = 10^-5 ^is added in the denominator to avoid division by zero. The smaller the GANGSTA score is, the larger is the structural agreement between the considered pair of proteins. Δ*SSE*, is defined as

ΔSSE=∑k=1NpairsSSE|dks−dkt|Max(∑k=1NpairsSSEdks,∑k=1NpairsSSEdkt),     (11)
 MathType@MTEF@5@5@+=feaafiart1ev1aaatCvAUfKttLearuWrP9MDH5MBPbIqV92AaeXatLxBI9gBaebbnrfifHhDYfgasaacH8akY=wiFfYdH8Gipec8Eeeu0xXdbba9frFj0=OqFfea0dXdd9vqai=hGuQ8kuc9pgc9s8qqaq=dirpe0xb9q8qiLsFr0=vr0=vr0dc8meaabaqaciaacaGaaeqabaqabeGadaaakeaacqqHuoarcqqGtbWucqqGtbWucqqGfbqrcqGH9aqpdaWcaaqaamaaqahabaWaaqWaaeaacqqGKbazdaqhaaWcbaGaee4AaSgabaGaee4CamhaaOGaeyOeI0Iaeeizaq2aa0baaSqaaiabbUgaRbqaaiabbsha0baaaOGaay5bSlaawIa7aaWcbaGaee4AaSMaeyypa0JaeGymaedabaGaeeOta40aa0baaWqaaiabbchaWjabbggaHjabbMgaPjabbkhaYjabbohaZbqaaiabbofatjabbofatjabbweafbaaa0GaeyyeIuoaaOqaaiabb2eanjabbggaHjabbIha4naabmaabaWaaabCaeaacqqGKbazdaqhaaWcbaGaee4AaSgabaGaee4CamhaaaqaaiabbUgaRjabg2da9iabigdaXaqaaiabb6eaonaaDaaameaacqqGWbaCcqqGHbqycqqGPbqAcqqGYbGCcqqGZbWCaeaacqqGtbWucqqGtbWucqqGfbqraaaaniabggHiLdGccqGGSaaldaaeWbqaaiabbsgaKnaaDaaaleaacqqGRbWAaeaacqqG0baDaaaabaGaee4AaSMaeyypa0JaeGymaedabaGaeeOta40aa0baaWqaaiabbchaWjabbggaHjabbMgaPjabbkhaYjabbohaZbqaaiabbofatjabbofatjabbweafbaaa0GaeyyeIuoaaOGaayjkaiaawMcaaaaacqGGSaalcaWLjaGaaCzcamaabmaabaGaeGymaeJaeGymaedacaGLOaGaayzkaaaaaa@8747@

where the sums run over the number of SSE pairs NpairsSSE
 MathType@MTEF@5@5@+=feaafiart1ev1aaatCvAUfKttLearuWrP9MDH5MBPbIqV92AaeXatLxBI9gBaebbnrfifHhDYfgasaacH8akY=wiFfYdH8Gipec8Eeeu0xXdbba9frFj0=OqFfea0dXdd9vqai=hGuQ8kuc9pgc9s8qqaq=dirpe0xb9q8qiLsFr0=vr0=vr0dc8meaabaqaciaacaGaaeqabaqabeGadaaakeaacqqGobGtdaqhaaWcbaGaeeiCaaNaeeyyaeMaeeyAaKMaeeOCaiNaee4CamhabaGaee4uamLaee4uamLaeeyraueaaaaa@3848@ considered for the structure alignment. The Euclidian distances dks
 MathType@MTEF@5@5@+=feaafiart1ev1aaatCvAUfKttLearuWrP9MDH5MBPbIqV92AaeXatLxBI9gBaebbnrfifHhDYfgasaacH8akY=wiFfYdH8Gipec8Eeeu0xXdbba9frFj0=OqFfea0dXdd9vqai=hGuQ8kuc9pgc9s8qqaq=dirpe0xb9q8qiLsFr0=vr0=vr0dc8meaabaqaciaacaGaaeqabaqabeGadaaakeaacqqGKbazdaqhaaWcbaGaee4AaSgabaGaee4Camhaaaaa@30F2@ and dkt
 MathType@MTEF@5@5@+=feaafiart1ev1aaatCvAUfKttLearuWrP9MDH5MBPbIqV92AaeXatLxBI9gBaebbnrfifHhDYfgasaacH8akY=wiFfYdH8Gipec8Eeeu0xXdbba9frFj0=OqFfea0dXdd9vqai=hGuQ8kuc9pgc9s8qqaq=dirpe0xb9q8qiLsFr0=vr0=vr0dc8meaabaqaciaacaGaaeqabaqabeGadaaakeaacqqGKbazdaqhaaWcbaGaee4AaSgabaGaeeiDaqhaaaaa@30F4@ in eq. (11) refer to the C_α _atoms in the SSE centers of the corresponding pairs of SSEs in source and target structures, respectively. A pair of aligned proteins with evanescent GANGSTA score posses structures that are identical on the employed resolution level of C_α _atom coordinates.

### Statistical significance of GANGSTA scores

To assess the quality of pairwise protein-structure alignments we use a method described by Ortiz et al. [50] and Vesterstrøm et al. [14] following the work of Levitt and Gerstein [54] and Abagyan and Batalov [62]. To estimate the statistical significance of GANGSTA scores, eq. (10), we calculate a *P*-value describing the probability to get a better GANGSTA score than observed when aligning unrelated structures. This *P*-value can be obtained by fitting a Type I extreme value distribution function (Gumbel distribution) on the GANGSTA score distribution resulting from pairwise structure alignments of unrelated proteins. The Gumbel distribution possesses the probability density function [63].

fG(x)=1bexp⁡(−(x−a)b)exp⁡[−exp⁡((a−x)b)],     (15)
 MathType@MTEF@5@5@+=feaafiart1ev1aaatCvAUfKttLearuWrP9MDH5MBPbIqV92AaeXatLxBI9gBaebbnrfifHhDYfgasaacH8akY=wiFfYdH8Gipec8Eeeu0xXdbba9frFj0=OqFfea0dXdd9vqai=hGuQ8kuc9pgc9s8qqaq=dirpe0xb9q8qiLsFr0=vr0=vr0dc8meaabaqaciaacaGaaeqabaqabeGadaaakeaacqWGMbGzdaWgaaWcbaGaem4raCeabeaakiabcIcaOiabdIha4jabcMcaPiabg2da9maalaaabaGaeGymaedabaGaemOyaigaaiGbcwgaLjabcIha4jabcchaWjabcIcaOmaalaaabaGaeyOeI0IaeiikaGIaemiEaGNaeyOeI0IaemyyaeMaeiykaKcabaGaemOyaigaaiabcMcaPiGbcwgaLjabcIha4jabcchaWjabcUfaBjabgkHiTiGbcwgaLjabcIha4jabcchaWjabcIcaOmaalaaabaGaeiikaGIaemyyaeMaeyOeI0IaemiEaGNaeiykaKcabaGaemOyaigaaiabcMcaPiabc2faDjabcYcaSiaaxMaacaWLjaWaaeWaaeaacqaIXaqmcqaI1aqnaiaawIcacaGLPaaaaaa@5D3B@

with parameters *a *and *b *for location and width, respectively. To fit the GANGSTA score distribution with the Gumbel probability density function the parameters *a *and *b *in eq. (15) need to be determined. Since protein-structure alignments are of higher quality for smaller GANGSTA scores, this part of the Gumbel distribution is more relevant for the fit than the tail at larger GANGSTA scores [50]. Therefore, we evaluated the probability to obtain GANGSTA scores *t *lower than *x*. The corresponding expression of the Gumbel distribution reads

PG(t≤x)=∫0xfG(t)dt=exp⁡[−exp⁡(a−xb)].     (16)
MathType@MTEF@5@5@+=feaafiart1ev1aaatCvAUfKttLearuWrP9MDH5MBPbIqV92AaeXatLxBI9gBaebbnrfifHhDYfgasaacH8akY=wiFfYdH8Gipec8Eeeu0xXdbba9frFj0=OqFfea0dXdd9vqai=hGuQ8kuc9pgc9s8qqaq=dirpe0xb9q8qiLsFr0=vr0=vr0dc8meaabaqaciaacaGaaeqabaqabeGadaaakeaacqWGqbaudaWgaaWcbaGaem4raCeabeaakiabcIcaOiabdsha0jabgsMiJkabdIha4jabcMcaPiabg2da9maapehabaGaemOzay2aaSbaaSqaaiabdEeahbqabaaabaGaeGimaadabaGaemiEaGhaniabgUIiYdGccqGGOaakcqWG0baDcqGGPaqkcqWGKbazcqWG0baDcqGH9aqpcyGGLbqzcqGG4baEcqGGWbaCcqGGBbWwcqGHsislcyGGLbqzcqGG4baEcqGGWbaCcqGGOaakdaWcaaqaaiabdggaHjabgkHiTiabdIha4bqaaiabdkgaIbaacqGGPaqkcqGGDbqxcqGGUaGlcaWLjaGaaCzcamaabmaabaGaeGymaeJaeGOnaydacaGLOaGaayzkaaaaaa@5CFB@

Eq. 16 can be transformed into a linear expression by applying the logarithm twice yielding

ln⁡(−ln⁡(PG(t≤x)))=ab−1bx.     (17)
 MathType@MTEF@5@5@+=feaafiart1ev1aaatCvAUfKttLearuWrP9MDH5MBPbIqV92AaeXatLxBI9gBaebbnrfifHhDYfgasaacH8akY=wiFfYdH8Gipec8Eeeu0xXdbba9frFj0=OqFfea0dXdd9vqai=hGuQ8kuc9pgc9s8qqaq=dirpe0xb9q8qiLsFr0=vr0=vr0dc8meaabaqaciaacaGaaeqabaqabeGadaaakeaacyGGSbaBcqGGUbGBcqGGOaakcqGHsislcyGGSbaBcqGGUbGBcqGGOaakcqWGqbaudaWgaaWcbaGaem4raCeabeaakiabcIcaOiabdsha0jabgsMiJkabdIha4jabcMcaPiabcMcaPiabcMcaPiabg2da9maalaaabaGaemyyaegabaGaemOyaigaaiabgkHiTmaalaaabaGaeGymaedabaGaemOyaigaaiabdIha4jabc6caUiaaxMaacaWLjaWaaeWaaeaacqaIXaqmcqaI3aWnaiaawIcacaGLPaaaaaa@4D4E@

The parameters *a *and *b *can now easily be estimated by a linear fit between the probability of GANGSTA scores *t *≤ *x *obtained from structure alignment of unrelated proteins and the corresponding probability P_G_(*t *≤ *x*) form the Gumbel distribution. Once we have determined *a *and *b*, we can calculate the mean

μ = *a *+ γ*b*,     (18)

where γ = 0.5772 is the Euler-Mascheroni constant and the standard deviation

σ=π6b     (19)
 MathType@MTEF@5@5@+=feaafiart1ev1aaatCvAUfKttLearuWrP9MDH5MBPbIqV92AaeXatLxBI9gBaebbnrfifHhDYfgasaacH8akY=wiFfYdH8Gipec8Eeeu0xXdbba9frFj0=OqFfea0dXdd9vqai=hGuQ8kuc9pgc9s8qqaq=dirpe0xb9q8qiLsFr0=vr0=vr0dc8meaabaqaciaacaGaaeqabaqabeGadaaakeaacqaHdpWCcqGH9aqpdaWcaaqaaiabec8aWbqaamaakaaabaGaeGOnaydaleqaaaaakiabdkgaIjaaxMaacaWLjaWaaeWaaeaacqaIXaqmcqaI5aqoaiaawIcacaGLPaaaaaa@386B@

of this distribution. Using the linear transformation z=x−μσ
 MathType@MTEF@5@5@+=feaafiart1ev1aaatCvAUfKttLearuWrP9MDH5MBPbIqV92AaeXatLxBI9gBaebbnrfifHhDYfgasaacH8akY=wiFfYdH8Gipec8Eeeu0xXdbba9frFj0=OqFfea0dXdd9vqai=hGuQ8kuc9pgc9s8qqaq=dirpe0xb9q8qiLsFr0=vr0=vr0dc8meaabaqaciaacaGaaeqabaqabeGadaaakeaacqWG6bGEcqGH9aqpdaWcaaqaaiabdIha4jabgkHiTiabeY7aTbqaaiabeo8aZbaaaaa@351E@ the probability in eq.(16) can also be interpreted as *z*-score

PG(X<z)=exp⁡[−exp⁡(π6z+γ)].     (20)
 MathType@MTEF@5@5@+=feaafiart1ev1aaatCvAUfKttLearuWrP9MDH5MBPbIqV92AaeXatLxBI9gBaebbnrfifHhDYfgasaacH8akY=wiFfYdH8Gipec8Eeeu0xXdbba9frFj0=OqFfea0dXdd9vqai=hGuQ8kuc9pgc9s8qqaq=dirpe0xb9q8qiLsFr0=vr0=vr0dc8meaabaqaciaacaGaaeqabaqabeGadaaakeaacqWGqbaudaWgaaWcbaGaem4raCeabeaakiabcIcaOiabdIfayjabgYda8iabdQha6jabcMcaPiabg2da9iGbcwgaLjabcIha4jabcchaWjabcUfaBjabgkHiTiGbcwgaLjabcIha4jabcchaWjabcIcaOmaalaaabaGaeqiWdahabaWaaOaaaeaacqaI2aGnaSqabaaaaOGaemOEaONaey4kaSIaeq4SdCMaeiykaKIaeiyxa0LaeiOla4IaaCzcaiaaxMaadaqadaqaaiabikdaYiabicdaWaGaayjkaiaawMcaaaaa@4F9E@

### Database search

For a database scan a reference structure is compared (aligned) with all sample structures in the database. In most applications the reference structure is also the *source *structure, i.e. the reference structure is smaller than the sample structure from the database. However, the reference structure can also be the *target *structure if the sample structure from the database is smaller than the reference structure.

To speed up database searches a pre-filter is applied to limit the search for proteins that match certain criteria. These involve the number of SSEs, the structure diameter (i.e. maximum distance between any pair of SSE measured between the Cα atoms in the SSE centers) and the number of SSEs in contact (based on Cα atom distances). A protein structure from the database (sample structure) is only considered for structure alignment if the corresponding pair of source and target structures fulfill the following three basic criteria. (i) The target structure has at most one α-helix or one β-strand less than the source structure. (ii) The structure diameter of the source structure should be at most twice as large as the diameter of the target structure. (iii) The source structure should have no more than twice as many α-helix or β-strand pairs in contact as compared to the target structure.

Additionally, for the computationally demanding second level of the method, the residue-based structure alignment step, a rough estimate for the contact map optimization is used. To estimate the contact overlap value *q*, eq. (9), we use a greedy-algorithm, which starts by finding the optimal offset (see Fig. 10) for the considered SSE pair yielding the largest number of contacts. Then the algorithm continues by finding the optimal offset for the pair having the second largest number of contacts and so forth. While the problem of finding a global optimal residue alignment cannot be solved with a such a local strategy, the estimated overlap values are in good agreement with optimal results. However, this estimate is sometimes up to 10,000 times faster than the method used for finding optimal structure alignments on the residue level as described above. Since we are using an estimated contact overlap *q*, eq. (9), the reported *P*-value for database scans is only an upper bound of the *P*-value for pairwise alignments.

### Protein-structure datasets

#### Non-redundant dataset of protein structures (SCOP40)

We used a non-redundant subset of the ASTRAL SCOP compendium [64] version 1.67 including only SCOP [48] domain structures with at most 40% sequence identity. The SCOP40 dataset can be downloaded from the ASTRAL webpage [65]. To guarantee an appropriate performance of GANGSTA all structures in the dataset contain at least two SSEs and have more than 30% of their residues in SSEs resulting in 7158 domain structures. This dataset is used for all database searches with the web version of GANGSTA.

#### Protein-structure datasets for statistical significance of classification tasks

From the SCOP40 dataset we generated two additional datasets. SAME_SF40 consists of 4982 random pairs of SCOP domain structures taken from the same SCOP superfamilies. The protein pairs involve 672 different SCOP domains taken from 113 different SCOP superfamilies belonging to 99 different SCOP folds. DIFF_SF40 consists of 88909 random pairs of SCOP domain structures where for each pair the proteins are taken from different SCOP superfamilies. This dataset of protein pairs involves 500 different SCOP domains from 317 different SCOP superfamilies belonging to 243 different SCOP folds. The domain lists of DIFF40 and SAME40 are shown in Tables S6 and S7 [see Additional file 1]. The list of the corresponding domain pairs can be provided on request.

### Four-Helix-Bundle dataset

This dataset comprises ten proteins belonging to four different folds and six different superfamilies in the SCOP classification scheme. Table S3 [see Additional file 1] shows the dataset of ten proteins and their SCOP annotations [48]. This dataset was used before in [44].

### TRAF dataset

The dataset consists of eight proteins that belong to two different folds in the all-β class of the SCOP database. Four proteins (PDB-IDs: 1czyA, 1kzzA, 1lb4, 1k2fA) belong to the "TRAF (TNF Receptor Associated Factor) domain-like" fold but are members of two different families: 1czyA, 1kzzA, and 1lb4 were taken from the "TRAF domain" family, 1k2fA belongs to the "SIAH" family. Four proteins (PDB-IDs: 1bmg, 1frtB, 1igtA, 1k8iA) of the TRAF dataset belong to the "C1 set domains" family of the "Immunoglobulin-like beta-sandwich" fold. This dataset was used before in [44].

### Rossmann dataset

The dataset consists of seven protein domains that contain Rossmann and Rossmann-like structure motifs according to CATH or SCOP classification schemes. The proteins (target structures) are listed in Fig. 7. All proteins have less than 40% sequence similarity.

### Fischer dataset

This dataset consist of ten protein-structure pairs introduced by Fischer et al. [66] and used by Novotny et al. [53]. Novotny added the last pair (1g61/1jdw). The PDB ids of the protein pairs are: 1bgeB/2gmfA, 1cewI/1molA, 1cid/2rhe, 1crl/1ede, 1fxiA/1ubq, 1ten/1hhrB, 1tie/4fgf, 2azaA/1paz, 2sim/1nsbA, 3hlaB/2rhe, 1g61/1jdw.

### Novotny dataset

This dataset consists of representative proteins from four different CATH [46] classes (classes: *mainly*-α, *mainly*-β, *mixed*-α-β, *few SSEs*) and was applied in a recent performance test by Novotny et al. [53]. The protein domains and their corresponding CATH classifications are listed in Table S5 [see Additional file 1]. The whole Novotny dataset and the benchmark results are available on [67].

## Authors' contributions

BK designed and carried out research and drafted manuscript.

PM designed and carried out research and drafted manuscript.

TSG discussed and applied GANGSTA methodology.

TS coordinated research and helped to draft manuscript.

EWK coordinated and designed research and drafted manuscript.

All authors read and approved this final version.

## Supplementary Material

Additional file 1 **Supplement Data**. Microsoft Word Document. Contains additional information on implementation details of the algorithm, additions to the analysis of the significance of the GANGSTA score, detailed datasets used for our tests, detailed listing of the clusters found for the Rossmann structure motif. Click here for file
