# Supplementary material for: Connectivity independent protein-structure alignment: a hierarchical approach
Source: BMC Bioinformatics. 2006 Nov 21;7:510. doi: 10.1186/1471-2105-7-510 (PMC1683948; doi:10.1186/1471-2105-7-510)
Supplement: Additional file 1 — Supplement Data. Microsoft Word Document. Contains additional information on implementation details of the algorithm, additions to the analysis of the significance of the GANGSTA score, detailed datasets used for our tests, detailed listing of the clusters found for the Rossmann structure motif. [file 1471-2105-7-510-S1.doc]

**Supplement Data**

# Connectivity independent protein-structure alignment: A hierarchical approach

Bjoern Kolbeck ^1 *^, Patrick May ^2,1 *^, Tobias Schmidt-Goenner ^1^, Thomas Steinke ^2^ and Ernst-Walter Knapp ^1§^

^1^ Macromolecular Modeling Group, Institute of Chemistry and Biochemistry, FU Berlin, Takustrasse 6, 14195 Berlin, Germany

^2^ Computer Science Research, Zuse Institute Berlin, Takustrasse 7, 14195 Berlin, Germany

*These authors contributed equally to this work

^§^Corresponding author

# Implementation Details

The protein structure alignment can be performed with a user-specified bias preferring sequential or non-sequential connectivity alignments, tuned by the term *Seq* in the objective function, eq. (6). The function *Seq(*$\vec{V}^{s},g^{st}$*)* is used to either favor (SB < 0) or disfavor (SB > 0) sequential alignments

$Seq(\vec{V}^{s},g^{st}) = SB \frac{\sum_{i=1}^{\left|V^{s}\right|-1} br(v_{i}^{s},v_{i+1}^{s},g^{st})}{\left|V^{s}\right|-1}$ (S1)

where $\vec{V}^{s} = (v_{1}^{s}, v_{2}^{s}, . . . v_{|V^{s}|}^{s})$ is the set of ordered vertices of the aligned source protein structure (*s*) and $\vec{V}^{t} = (v_{1}^{t}, v_{2}^{t}, . . . v_{|V^{t}|}^{t})$ the set of ordered vertices of the target protein structure (*t*). The function

$br(v_{i}^{s},v_{i+1}^{s},g^{st})=\left\{\begin{matrix} 1, \text{if for} \left[v_{k_{i}}^{t} = g_{V}(v_{i}^{s}) \text{and} v_{k_{i+1}}^{t} = g_{V}(v_{i+1}^{s}) \right] \text{follows} k_{i} > k_{i+1} \\ 0, \text{else} \end{matrix}\right.$ (S2)

detects how often the connectivity of the target structure *(t)* differs from the connectivity of the source structure *(s)*. The function *g_V_* in eqs. (1) and (2) maps vertices from the aligned (target) structure on corresponding vertices of the source structure. *SB*, eq. (S1), is a parameter controlling the strength of the bias. Positive *SB* values favor structures aligned with sequential SSE connectivity while negative *SB* values disfavor such alignments.

The SSE length penalty L, used in eq. (6), is calculated according to the following expressions

$\begin{matrix} \Delta L(v,g^{st})=\left|f_{L}^{V}(v)-f_{L}^{V}(g_{V}(v))\right| \\ LP(v,g^{st})=\left\{\begin{matrix} 0.03 & \text{if} \Delta L(v,g^{st})>3 \\ 0.01 & \text{if} 0>\Delta L(v,g^{st})>-4 \\ 0.1 & \text{if} \Delta L(v,g^{st})<-3 \\ 0, & \text{otherwise or if }v\text{ is a gap} \end{matrix}\right. \\ L(V^{S},g^{st})=\sum_{v\in V^{S}} LP(v,g^{st}) \end{matrix}$ (S3)

with ΔL giving the length difference between matched SSEs and LP the individual penalty for each SSE pair depending on ΔL. L is the sum of penalties over all SSEs.


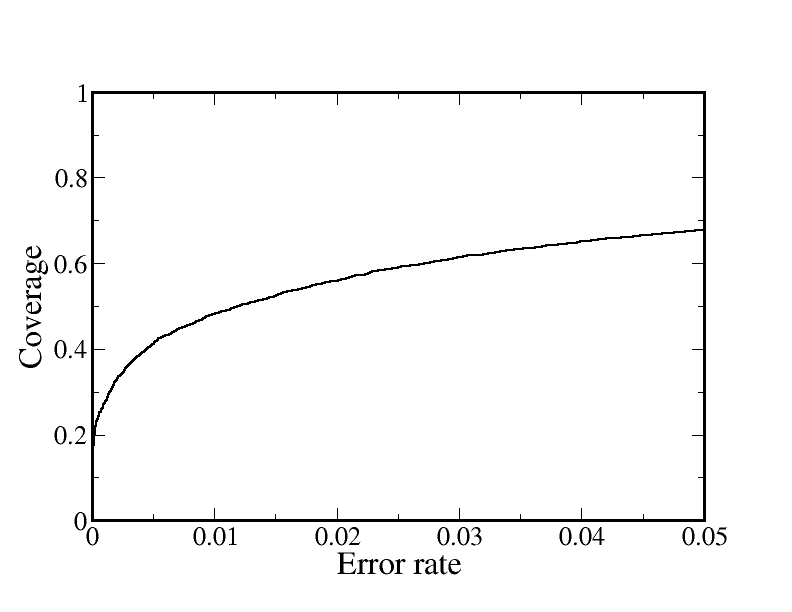


## Figure S1 – Coverage versus error rate plot for GANGSTA *scores*

For a given P-value threshold, we calculated the percentage of true positives (i.e. percentage of alignment for pairs from the same SCOP {Murzin, 1995 #6} superfamily with a GANGSTA *Score* detected for a given error rate (i.e. number of false positives per query).

## TABLE S1 - GANGSTA performance

Runtimes for pairwise protein structure alignments and database scans.

| source protein | target protein | CPU time including IO |
| --- | --- | --- |
| 2uagA1  2uagA1  2uagA1  1ziwA  2uagA1 | 1gkuB1  1dhs  1cjcA2  2a0zA  database scan | 0.579s  0.707s  0.575s  19.06s  13m32s |

TABLE S2 – Parameters of the objective function, eq. (6), used for the Genetic Algorithm

| **parameter** | value |
| --- | --- |
| w_C_ | 0.6 |
| w_O_ | 0.4 |
| GP:  gap penalty | 0.11 * Ngap (Ngap: number of ignored SSE in source structure) |
| SB: SSE connectivity | parameter can be set by user,  default value SB = 0 (no preference) |
| ε | 10^-9^ |

**TABLE S3 – Four-Helix-Bundle dataset**

| **protein** | **SCOP fold** | **SCOP superfamily** |
| --- | --- | --- |
| 2hmzA | 47161 | 47188 |
| 2ccyA | 47161 | 47175 |
| 256bA | 47161 | 47175 |
| 1bbhA | 47161 | 47175 |
| 1le2 | 47161 | 47162 |
| 3inkC | 47265 | 47266 |
| 1bgeB | 47265 | 47266 |
| 1rcb | 47265 | 47266 |
| 1aep | 47856 | 47857 |
| 1flx | designed **^a^** | designed **^a^** |

**^a^** SCOP category for a selection of artificial protein structures.

## TABLE S4 – Rossmann dataset

| **SCOP id** | **CATH-id** | **CAT ^a^** | **CATH ^b^** |
| --- | --- | --- | --- |
| 1cjcA2 | 1cjcA01 | 3.40.50 | 3.40.50.720 |
| 1rqlA_ | 1rqlA01  1rqlA02 | 3.40.50  1.10.164 | 3.40.50.1000  1.10.164.10 |
| 1geeA_ | 1geeA00 | 3.40.50 | 3.40.50.720 |
| 1dhs__ | 1dhs000 | 3.40.910 | 3.40.910.10 |
| 1dih_1 | 1dih001 | 3.40.50 | 3.40.50.720 |
| 1f0kA_ | 1f0kA01  1f0kA02 | 3.40.50  3.40.50 | 3.40.50.2000  3.40.50.2000 |
| 1f8yA_ | 1f8yA00 | 3.40.50 | 3.40.50.1810 |

^a^ Class-Architecture-Topology code according to CATH.

^b^ Class-Architecture-Topology-Homologous Superfamily code according to CATH.

## TABLE S5 – Novotny dataset (from Novotny et al. {Novotny, 2004 #45}) with CATH classification

For all proteins the CATH class, the CATH topology classification, the number of different superfamilies per topology level in the dataset, and the CATH domain identifier are listed.

| Class | CAT^a^ | No H^b^ | CATH entries |
| --- | --- | --- | --- |
| mainly-α | 1.10.40 | 2 | 1rlr001 1yfm003 1furA03 1auwA03 1jswB03 1hylC03 1i0aA03 |
| mainly-α | 1.10.164 | 3 | 1aq6A02 1c3uA02^c^ 1fezA02 1jud002 1zrn001 |
| mainly-α | 1.25.10^d^ | 3 | 1b3uA00 1bk6A00 1gcjA00 1ialA00 1ibrB00 1qbkB00 2bct000 |
| mainly-β | 2.30.110 | 2 | 1ci0A00 1dnlA00 1ejeA00 1i0rA00 |
| mainly-β | 2.40.100 | 1 | 1a33000 1awgA00 1cynA00 1dywA00 1ihgA01 1lopA00 1qngA00 1qoiA00 2rmcA00 |
| mainly-β | 2.100.10 | 3 | 1c3kA00 1ciy002 1jacA00 1jotA00 1dlc003 1vmoA00 |
| mixed-α-β | 3.10.50 | 2 | 1bkf000 1grj002 1pbk000 1rot000 1yat000 |
| mixed-α-β | 3.40.91 | 3 | 1bhmA00 1cfr000 1d2iA00 1fokA03 |
| mixed-α-β | 3.70.10 | 3 | 1axcA00 1b77^f^ 1czd^f^ 1dmlAa00 1ge8A00 1plq000 |
| few SSEs | 2.40.20 | 1 | 1b2iA00 1ceaA00 1kdu000 1kiv000 1krn000 1pk4000 1pmlA00 5hgp^g^ |

^a^Class-Architecture-Topology code according to CATH.

^b^Number of homologous superfamilies (H level in CATH) of the topology level.

^c^Has changed CATH topology from L-2-haloacid dehalogenase, domain 2 to fumarase C chain A, domain 2.

^d^1.25.30 in the original Novotny set. All domains have moved to 1.25.10.

^e^3.10.70 in the original Novotny set. All domains have moved to 3.10.50.

^f^Not classified anymore.

^g^Not in the PDB anymore.

**TABLE S6.** **672 SCOP domains of the dataset DIFF_SF40.**

1v25a_ 1sgpe_ 1a0ca_ 1nh2a1 1h7wa2 1t3ta7 1l8ya_ 2mev1_ 1tqia1 1kjqa2

1jk7a_ 1qqp1_ 1hdoa_ 1ukka_ 1ufya_ 2scpa_ 1dova_ 1i24a_ 1kbza_ 1gega_

1dlea_ 1nyaa_ 1t64a_ 1s0aa_ 1f9za_ 1ayaa_ 1nvmb1 1o7fa3 1nw6a_ 1s6ca_

1bev1_ 1vlwa_ 1qkia1 1f3ub_ 1eny__ 1bjna_ 1fo8a_ 1e5sa_ 1ogla_ 1s6ia_

1q8ba_ 1axca2 1uaya_ 1omza_ 1rkxa_ 2tpt_1 1htp__ 1eoka_ 1elva1 1hb6a_

1d5ta2 1ssxa_ 1uspa_ 1vl0a_ 1teaa1 1q8ha_ 1h9sa1 1ehia1 1s83a_ 1qb2a_

1pf5a_ 1azza_ 1i4na_ 1enp__ 1p22a1 1df9a_ 1exma2 1o94a1 1p7ia_ 1orra_

1j0ma2 1ld8a_ 1psza_ 1gdna_ 2trxa_ 1vj1a1 1booa_ 2sak__ 1r17a1 1hyha2

1lshb_ 1lrza2 1rwha2 1h6da1 1k8kf_ 1rwza1 1p57b_ 1p5dx4 1e0ta3 1lo7a_

1ju5a_ 1b77a1 1r8na_ 1t35a_ 1r0va1 1nm3a1 1k61a_ 1dgma_ 1dl2a_ 1dgua_

1qyca_ 1ofda2 1lqla_ 1ner__ 1a3wa3 1lmb3_ 1iyu__ 1e7la2 1m45a_ 1bio__

2cuaa_ 2hlca_ 1e3oc2 1k99a_ 1s4na_ 1p0ka_ 1h5qa_ 1ooea_ 1ctaa_ 1iz5a1

1k6ja_ 1adoa_ 1miwa1 1rtra_ 1utg__ 1jtva_ 1v4ea_ 1avac_ 1tk5a2 1e7wa_

1j3va1 1j3da_ 1lkka_ 1s95a_ 1un8a4 1d2pa4 2gsaa_ 1b6ra2 1qy6a_ 1ij5a_

1o6za2 1obfo1 1vl8a_ 1l6wa_ 1dqia_ 1r26a_ 1jqba1 1lrza3 1akha_ 1nyed_

1mija_ 1dv1a2 1c4ra_ 1i36a1 1plq_1 1t4ba1 1okqa2 1i3oe_ 1ud9a1 1q27a_

1ri7a1 1geea_ 1r17a2 1j33a_ 1i5pa3 2cbla3 1j3wa_ 2dkb__ 1syra_ 1seia_

1n7oa2 1r69__ 1arb__ 1cnv__ 1p42a1 1qqra_ 1sffa_ 1h30a2 7mdha2 1iz0a1

1hzta_ 1dmla1 1qba_3 1orja_ 1j5va_ 1ego__ 1qrva_ 1fjha_ 1itxa1 1f74a_

1ek6a_ 1i2ta_ 1jbaa_ 1ti6b1 1ic8a2 1auib_ 1ub0a_ 1qjpa_ 1vnd__ 1d2sa_

1r0va2 1vk3a4 1thqa_ 1q3xa1 1hxha_ 2cmd_2 1spxa_ 2sli_1 1lara1 1v54b1

1c3ma_ 2dap_1 1d2oa1 1uhna_ 1geqa_ 1nvma2 1e3ma3 1vhoa1 1h30a1 1bg1a1

1dosa_ 1clia2 1nar__ 1autc_ 1nn7a_ 1t5hx_ 1gt0d_ 1t3ea3 1plq_2 1ep3a_

1p4ca_ 1neka2 1ca1_2 1kq1a_ 1p9la1 1fo3a_ 1dcea1 1xfla_ 1l7da2 1db3a_

1p1ja1 1bx4a_ 1qu9a_ 1j34c_ 1veta_ 1fu6a_ 1nexb1 1cb8a2 1a53__ 1e31a_

1puoa1 1gp6a_ 1qv0a_ 1rh1a2 1vkna1 1ouwa_ 1iloa_ 1jx7a_ 1rpya_ 1avwb_

1p3ca_ 1klih_ 1uufa1 1g4us2 1m61a1 1ciy_3 1v1aa_ 1v8aa_ 1woua_ 1krea_

1h9ka1 1gqna_ 1vk3a3 1exra_ 1k8ma_ 1emua_ 1bmlc3 1ff9a1 1sb8a_ 1pufb_

1p42a2 1fjgd_ 1l5oa_ 1fyc__ 1gvkb_ 1fbl_1 1ifwa_ 1gsoa2 1utxa_ 1gps__

1rzsa_ 1pq4a_ 1jd1b_ 1aiu__ 1vjpa1 1a7s__ 1d4ta_ 1pgja1 2plda_ 1ta3a_

1wdcb_ 1fx7a2 1a9xa3 1ne9a2 1st6a4 3kvt__ 1jkza_ 1mbma_ 1qkra_ 1f60a2

2pf1_2 1j7qa_ 2lefa_ 1jj2u_ 1cii_1 1kk1a2 1on2a2 1px0a_ 1jaka1 1khda1

1nh2a2 1dmla2 1ps9a1 1edt__ 1rwza2 1cx4a1 1n2za_ 1tiza_ 1snya_ 1mdba_

1hk9a_ 1jlna_ 1uhsa_ 1qj8a_ 1vhea1 1k8kd1 1sena_ 1f6ya_ 1c7va_ 1hn0a3

1t3ta6 1m9ua_ 1hkka1 1kl1a_ 1vhca_ 1efub2 1rkd__ 1o5ka_ 1u1ia1 1c3pa_

1bs0a_ 1m61a2 1brz__ 1pz7a_ 1uura1 1t1da_ 1q8ia2 1j5pa4 1wota_ 1jnra2

1qsga_ 1jfka_ 1rqja_ 1dcs__ 1tgoa2 1iow_1 2pvba_ 1sra__ 1nnga_ 1jyra_

1pg4a_ 1b16a_ 1t2da2 1ngvb_ 1l1ja_ 1chua2 1lsha3 3pmga4 1t01a1 1v39__

1g60a_ 1ojua2 1cx4a2 1m7ya_ 1omwa1 1n67a1 1pjza_ 1gvza_ 1t01a2 2ae1__

1axca1 1lq9a_ 1o14a_ 1oyb__ 6ldh_2 1uby__ 1st6a3 1agre_ 1f9ma_ 1vfga1

1ez4a2 1htjf_ 1q3ea_ 1i50c2 1gen__ 1amua_ 1wba__ 1bdb__ 1hbka_ 1lvmb_

1uoua1 1l4db_ 1ipaa2 1ne9a1 1fjsa_ 1i3za_ 1agja_ 1cyx__ 1luia_ 1bw5__

1pk6a_ 1toaa_ 1q7za1 2bby__ 1ejga_ 1oaa__ 1h6ga2 1ub3a_ 1e4ea1 1k9aa2

1jnda1 1w2ya_ 1ujpa_ 1b35a_ 1bdo__ 1no5a_ 1iapa_ 1cyda_ 1fc4a_ 1pl7a1

1qqp3_ 1hl2a_ 1o91a_ 1r6da_ 1ghk__ 1pf0a_ 1p15a_ 1tlta1 1fmca_ 1f8fa1

1onra_ 1lcya2 1bu8a1 2mev3_ 1d8ja_ 1g5ba_ 1tfe__ 1o89a1 1lab__ 1saya2

1iz5a2 1v64a_ 1okqa1 1q45a_ 1eq2a_ 1hq1a_ 1vyra_ 1vj0a1 1ewqa3 1qlaa2

2sas__ 1nrva_ 1i6ua_ 1g73d_ 1llda2 1bdfa2 1h2ba1 1n67a2 1ckta_ 1juba_

1h75a_ 1vm7a_ 1jh3a_ 1edqa2 1st6a5 1jvba1 1puoa2 1iqaa_ 1mil__ 1gvfa_

1q7ca_ 1vm6a1 1st6a6 1n4na_ 1cgha_ 1gr0a1 1ojxa_ 1q9ia2 1h6ga1 2tnfa_

1w3ia_ 1fq0a_ 1lc0a1 1vkza2 1t6la2 1gz0a2 1f1sa3 1dg6a_ 1af7_2 1bhp__

1nowa1 1pk6c_ 1b0na2 1v0da_ 1mla_1 1kola1 1muwa_ 1fo5a_ 1m39a_ 1odma_

1k2wa_ 1uc7a_ 1gu7a1 1oi2a_ 1p4ta_ 1c3ha_ 1uz5a3 1s2da_ 1vc4a_ 1pvc1_

1fjla_ 1l1sa_ 1e3oc1 1nxca_ 1ebfa1 1r5ya_ 1o69a_ 1vzia1 1qzxa2 1ji6a3

1uura3 1lsha2 1fqia_ 1a79a1 1ufka_ 1r6la1 1q1na1 1heta1 1vk4a_ 1goia2

1ayj__ 1se0a_ 1ud9a2 1e6ua_ 1aly__ 1v82a_ 1tnra_ 1r73a_ 1p1xa_ 1b35b_

1a49a3 1f3ua_ 1k8kd2 1njka_ 1g9la_ 1aba__ 1d1la_ 1nmra_ 1ih7a2 1qada_

1kxga_ 1pii_2 1jxca_ 1kfwa1 1hl9a2 1iq8a1 1i5za2 1b7go1 1e3ha2 1omra_

1kbia1 1t1ra2 1hyea2 1eq9a_ 1oxna_ 1vj2a_ 1b77a2 1nxqa_ 1ngva_ 1ja9a_

1ohwa_ 1mdoa_ 1st6a7 1dcza_ 1nsj__ 1vg0a2 1lfb__ 1b9ha_ 1adr__ 1s5ua_

1dpga1 2oata_ 1pkla3 1viza_ 1qora1 1ky9a2 1t6la1 2shpa1 1pufa_ 1d2ka1

1gv1a2 1hdca_ 1gz6a_ 1bg1a3 1sr9a2 2pgd_1 1aym3_ 1fs1a1 1b8pa2 1n7ka_

1v8ga1 1v3ua1 1o5la1 2ebn__ 1aym1_ 1qopa_ 1o5ia_ 1lci__ 1vhna_ 1jd5a_

1f8ya_ 1l9ka_ 1vh6a_ 1r0ka2 1kte__ 1sota2 1dih_1 1qipa_ 1rcua_ 1n5da_

1rj8a_ 1gy8a_ 1d3ga_ 1qhua1 1u4ba2 1shya_ 1rqba2 1iy8a_ 1hxn__ 1svpa_

1to3a_ 1auia_ 1ne6a2 1e5ka_ 1xeaa1 1e3ha3 1h9sa2 1nm2a1 1pa1a_ 1h3fa2

1j9ba_ 1pii_1 1vetb_ 1aisa1 1itva_ 1ttza_ 1o7fa2 1h1da_ 1ou5a1 1lyva_

1x8ma_ 1efub4

**TABLE S7**. **500 SCOP domains of the dataset SAME_SF40.**

1dvoa_ 1gpr__ 1rzhl_ 1ttea1 1h7wa2 1dtx__ 1n71a_ 1lj8a3 1uapa_ 1a0i_2

1gmua2 1ji1a3 1guxb_ 1k92a1 1qo0d_ 1pmma_ 1jgta1 1k8kc_ 1p97a_ 1shwb_

1xxaa_ 1klxa_ 1ikop_ 3ulla_ 1qwda_ 1pz7a_ 1xeaa2 1j7xa_ 1vi2a2 1g2qa_

1t1da_ 1drk__ 1u3da1 1n91a_ 1g2ta_ 1i1rb_ 1fgua1 1qba_2 1etea_ 1nr0a2

1jlva2 1saya1 1q12a1 1p6va_ 1n1ba2 1mkya3 1lb2b_ 1usma_ 1cjwa_ 1m5ya3

1iqva_ 1t2da2 1pls__ 1d3ya_ 1tocr2 1qzma_ 1qama_ 1ogla_ 1q8ba_ 1l8wa_

1j4aa2 1jkxa_ 1chd__ 3pmga4 1ni3a1 1sdwa1 2pia_2 3mbp__ 1vjua_ 1j9ma_

1jlva1 1elva1 1ao0a2 1t3ea2 1uspa_ 1vrta1 1k8ke_ 1ka1a_ 1l7da1 1azza_

1i4na_ 1jr7a_ 1n57a_ 1tdza2 1kzla2 2pgd_2 5ruba2 1fjgm_ 1j0ma2 1qisa_

1ld8a_ 1mwwa_ 1ewqa4 1a79a2 1lla_3 1qxna_ 1ouoa_ 1fw8a_ 1mjha_ 1j55a_

1pwt__ 1bgf__ 1duvg1 1imhc1 1qlac_ 1h3ga2 1jvwa_ 1osya_ 1tvla_ 1pv9a1

1t11a1 1h6ga2 1e3ma1 1nzja_ 1ac5__ 1fkma2 1g55a_ 1vdka_ 1ptma_ 1fx8a_

1lbva_ 1su4a2 1pfba_ 1ujpa_ 1bdo__ 1t5oa_ 1mqva_ 1al3__ 1g6xa_ 1qlaa1

1r6la2 1wdkc1 1nofa1 6mhta_ 1k61a_ 1gsia_ 1onha_ 1g0u1_ 1qgva_ 1t3ta5

1q02a_ 1doi__ 1aoa_1 1h2sa_ 1mhna_ 1r1ta_ 1bu8a1 1v7mv_ 1tr0a_ 1oboa_

1jvaa3 1ptf__ 1kr7a_ 1fsea_ 1bw0a_ 1o75a1 1ccwb_ 2cuaa_ 1n26a3 1x7fa1

1iz5a2 1dd9a_ 1mnaa_ 1pfja_ 1si2a_ 1qz8a_ 1m06g_ 1bt3a_ 1b4ba_ 1cuk_1

1h5qa_ 1aisb1 1ix9a1 1qlaa2 1su8a_ 1q1ra2 1ig6a_ 1gc0a_ 1hw6a_ 1tuea_

1jli__ 1vjza_ 1iqza_ 1bdfa2 1rqga2 1jtva_ 1cs6a2 1smpi_ 1fyhb2 1okia2

1juba_ 1gqea_ 1r3ea2 1t3qb1 1q2ya_ 1sr4a_ 1hs6a1 1i4ga1 1cot__ 1o1za_

1b6ra2 2gsaa_ 1g0uf_ 1p5va1 1b3ta_ 1i1ga2 1fxkc_ 1o70a2 1t95a2 1dhn__

1mil__ 1floa1 1rrma_ 1nzya_ 1h67a_ 1gd0a_ 1rsoa_ 1h4ra1 1qpma_ 1opd__

1s6da_ 1dq3a2 1ohga_ 1k32a4 1f2qa1 1l8na2 1uema_ 1pmaa_ 1mg7a2 1jfma_

1q9ia2 1jdra_ 1o51a_ 1ghea_ 1k66a_ 1smva_ 1jl1a_ 1fgja_ 1h7ca_ 1qasa1

1um8a_ 1c3d__ 1ueba2 1hgxa_ 1pda_1 1rwha3 1prxa_ 1t3na2 1i50h_ 1ku9a_

1r0wa_ 1sj8a2 1bvsa1 1jroa4 1ln1a_ 1n12a_ 1ia9a_ 1qora2 1ssqa_ 1bqv__

1sxjb1 1tvda_ 1e6ba1 1vcc__ 1f14a1 1imja_ 1kzyc2 1noga_ 1umna_ 1cxya_

1goia3 1bkb_2 1ix2a_ 1twda_ 1p5dx3 1sq9a_ 1g66a_ 1ve9a2 1nd6a_ 1qhoa2

1vdua_ 1jcua_ 1fjgp_ 1qjpa_ 1fjgq_ 1s1qa_ 1moga_ 1vnd__ 1cqxa3 1ep3b1

1ji2a2 1c3ha_ 1enfa2 3thia_ 1mkfa_ 1m55a_ 1xvaa_ 1je8a_ 1hlva1 1usla_

1nh8a1 1gsma1 1di6a_ 1nx4a_ 1t4wa_ 1ncu__ 1feca2 1hcra_ 1onea1 1nhp_3

1nlxa_ 1gvea_ 1ui5a2 1s2xa_ 1uura3 1n8ja_ 1nz0a_ 1ufka_ 2u2fa_ 1j0aa_

2bce__ 2nlra_ 1vbfa_ 1lcya1 1rq0a_ 1j6ua1 2fmr__ 1u0ja_ 1gh7a3 1dosa_

1kkha2 1jpya_ 1b0ua_ 1n10a2 1aly__ 1uvqb2 1v62a_ 1cd1a2 1dz1a_ 1q44a_

1ekta_ 1qwta_ 1mdah_ 1g1ka_ 1k3ra1 1l7da2 1a44__ 1mpya2 1am2__ 1q3qa2

1ewqa2 1fnma4 1bx4a_ 1nw9b_ 1v92a_ 1uc8a1 1r0tb_ 1hssa_ 1cpza_ 1mvfd_

1kbia1 1eova2 1h7sa2 1dq3a3 1mvea_ 1leha1 1ogca_ 1uf3a_ 1jj2d_ 1kt7a_

1h3na1 1ngva_ 1mo9a1 1a8l_2 1nsj__ 1t95a1 1boua_ 1gpea2 1htta1 1eqfa1

1cmxa_ 1gv2a2 1ihga1 1d0qa_ 1zpda1 1o54a_ 1nat__ 1hp1a1 1efpa1 1ny5a1

1l9va2 1b8aa2 1dcqa2 1ihma_ 1eca__ 1eupa_ 1k92a2 2shpa1 1jmva_ 1d2ka1

1ozja_ 1hbna2 1g6ha_ 1i17a_ 1g5ha2 1vk3a3 1u5ta1 1p6pa_ 1emua_ 2ezh__

1bfd_2 1cc1l_ 1o9ya_ 1f2ea1 1o6aa_ 1nxma_ 1onwa1 1j79a_ 1f00i2 1ehia2

1d7ya1 1o5la1 1efdn_ 1rzsa_ 1tzpa_ 1poxa2 1qf5a_ 1iuja_ 1lnsa2 1gk8a2

1ggxa_ 1b34b_ 1szna1 1jaja_ 1wdcb_ 1ugpb_ 1dv5a_ 1rzta3 1gska3 1a9xa3

1n9la_ 1repc2 1n0ua3 1r89a3 1qxoa_ 1r7ja_ 1jx4a1 1fvka_ 1sur__ 1by2__

1u0sa_ 1mbma_ 1eswa_ 1gtra2 1u4ba2 1hbna1 1jyaa_ 1k9vf_ 1u0ka1 1cm9a_

1to3a_ 1u55a_ 1ixma_ 1xo1a1 1ddga1 1ulea_ 1qzna_ 1repc1 1ash__ 1ibia2

1v54c_ 1itza3 1q74a_ 1ne6a2 1bn8a_ 1jmja_ 1am7a_ 1oy5a_ 1v0wa2 1mwxa2

1nh2a2 1vmoa_ 1h3fa2 1u0ma1 1b6e__ 1tf5a4 1i2ma_ 1l0lb2 1j77a_ 1oqka_

1mdba_ 1tzyb_ 1wdna_ 1t0qc_ 1jfxa_ 1p22a2 1t2da1 1e7ka_ 1stfi_ 1mxa_3

**TABLE S8 -** **Structure alignments with 2uagA1 as reference protein clustered according to SSE connectivity.**

The protein domains have the following labels:

- # Structures that are classified as a Rossmann fold [11] in CATH [7]
- * Structures that are classified as Rossmann fold or Rossmann-like fold in SCOP
- + Structures that belong to the “PLP-dependent transferases” fold in SCOP .

No label indicates that the domain is not classified as Rossmann-fold in both CATH and SCOP.

| **SSE connectivity of aligned structures (44 different clusters)** | **aligned protein domains** |
| --- | --- |
| 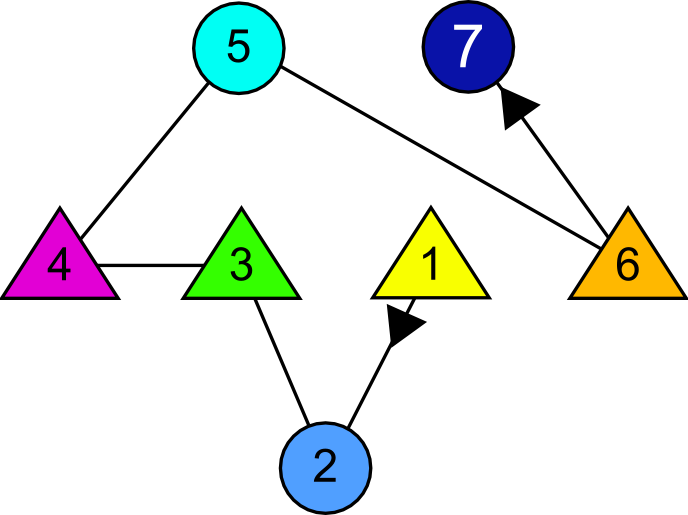  Same connectivity as 2uagA1 | 53 structures: 2uagA1, 1ff9A1, 1ngvB, 1jw9B, 1m2kA, 1q7eA, 1gycA, 1lssA, 1t1rA2, 1obbA1, 1rkd, 1k6jA, 1nw3A, 1id1A, 1iz0A2, 1js1X2, 1a9xA3, 1o94A, 1mjfA, 1qmgA2, 1v3nA2, 1f8fA2, 1b93A, 1vj5A1, 1uxnA, 1j5vA, 1k75A, 1u2zA, 1bg6_2, 1q37A, 1m6yA2, 1o20A, 1h2bA2, 1pqwA, 1gsoA2, 1duvG2, 1h1dA, 1lnqA1, 1dv1A2, 1o4wA, 1mulA, 1lj8A4, 1xvaA, 1ipaA1, 1meoA, 1aco_2, 1vj0A2, 1othA2, 2cmd_1, 1qorA2, 1te2A, 1vlnA, 1jscA |
| 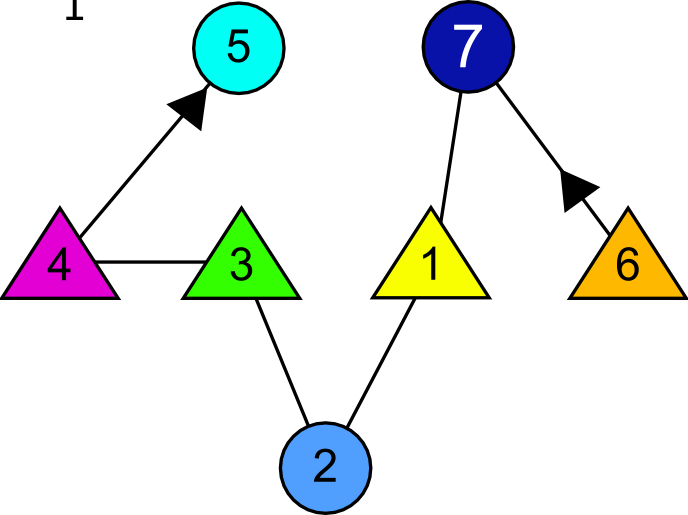 | 11 structures: 1rqlA#, 1dih_1*, 1qdlB#, 1dhs*, 1gyiA, 1f8yA#, 1qq5A#, 1uxoA, 1t35A, 1bmtA2#, 1pswA# |
| 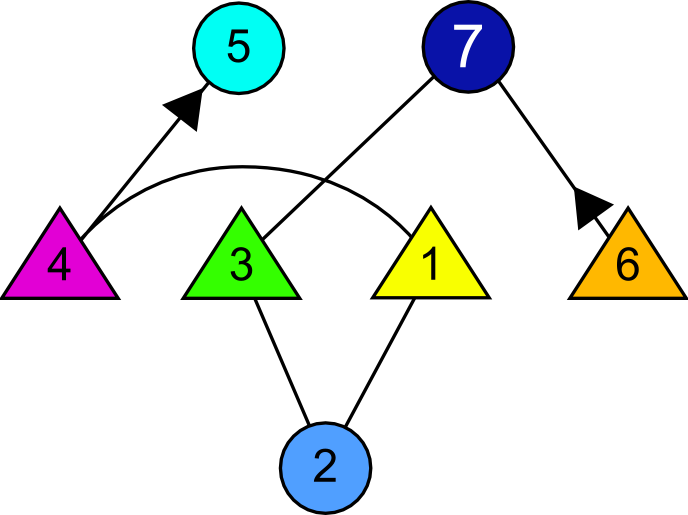 | 9 structures: 1gz6A,* 1cjcA2*, 1iy8A*, 1spxA*, 1geeA*, 1h7wA4*, 1ja9A*, 1fmcA*, 1r6dA* |
| 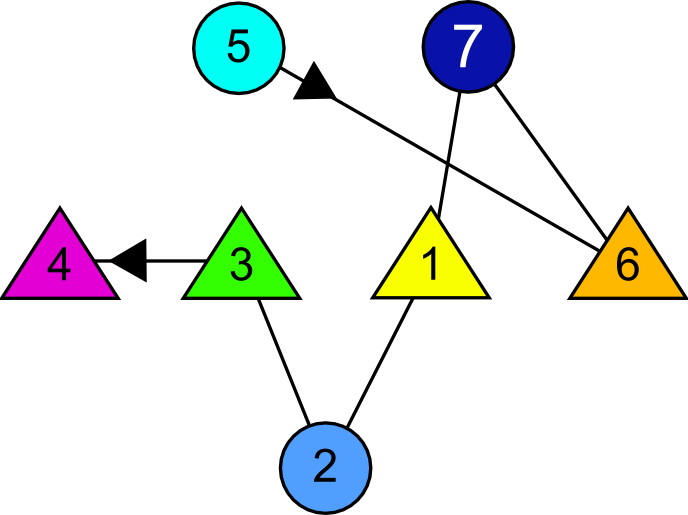 | 6 structures: 1gu7A2*, 1ibjA+, 1ou0A, 1uu1A+, 1mv8A3*, 1nw6A# |
| 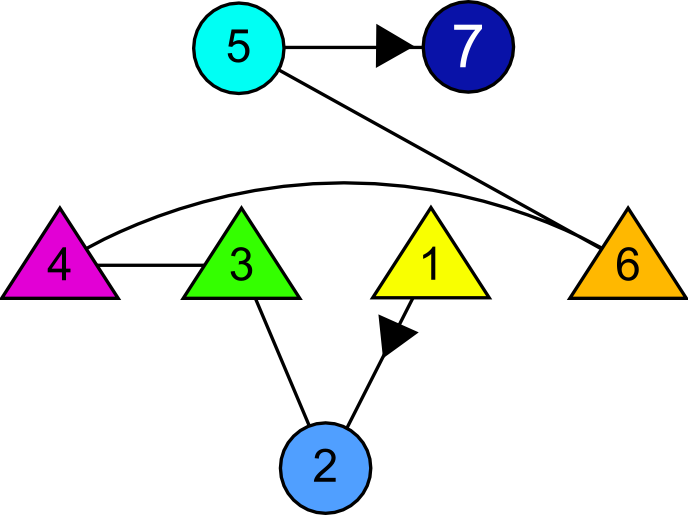 | 4 structures: 1f0kA#, 1npyA2*, 1jqbA2*, 1lsuA* |
| 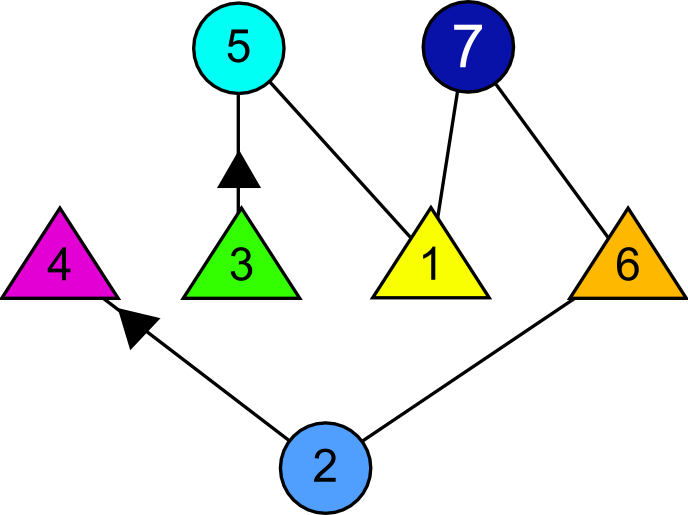 | 4 structures: 1oaa*, 2ae1*, 1ek6A*, 1b16A* |
| 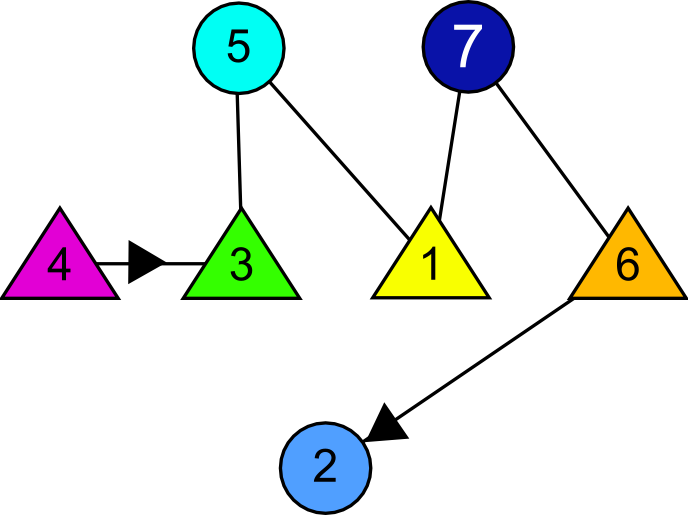 | 4 structures: 1p9lA1#, 1nat#, 1a2oA1#, 1j5pA4* |
| 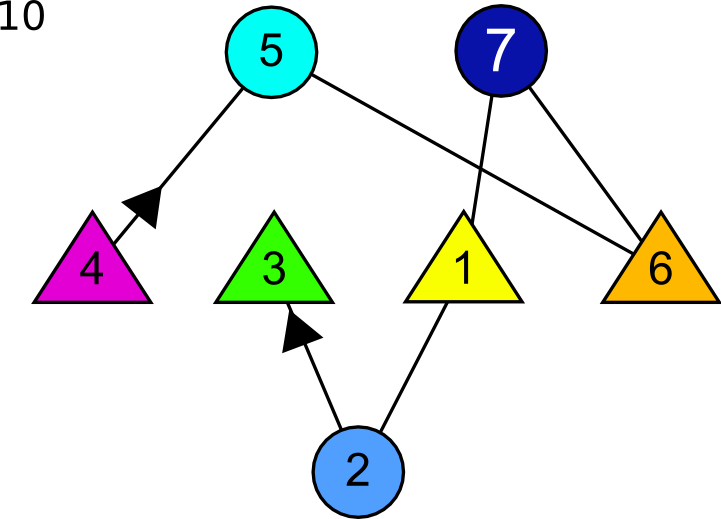 | 3 structures: 1q0vA*, 1hv8A1#, 1a1vA1# |
| 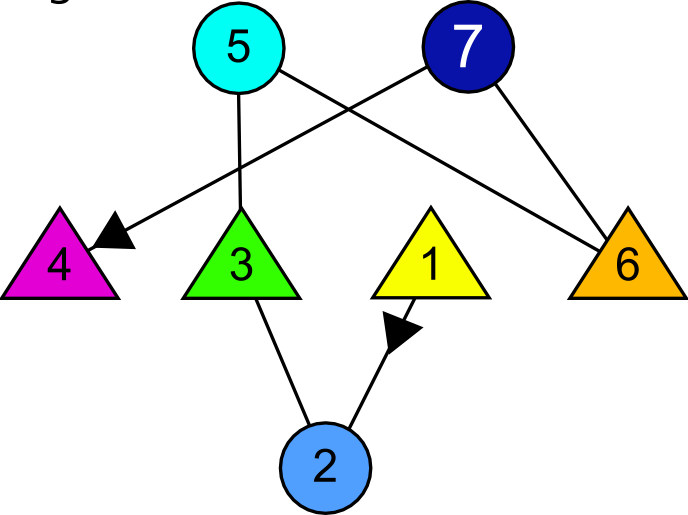 | 2 structures: 1o69A+, 1mb3A# |
| 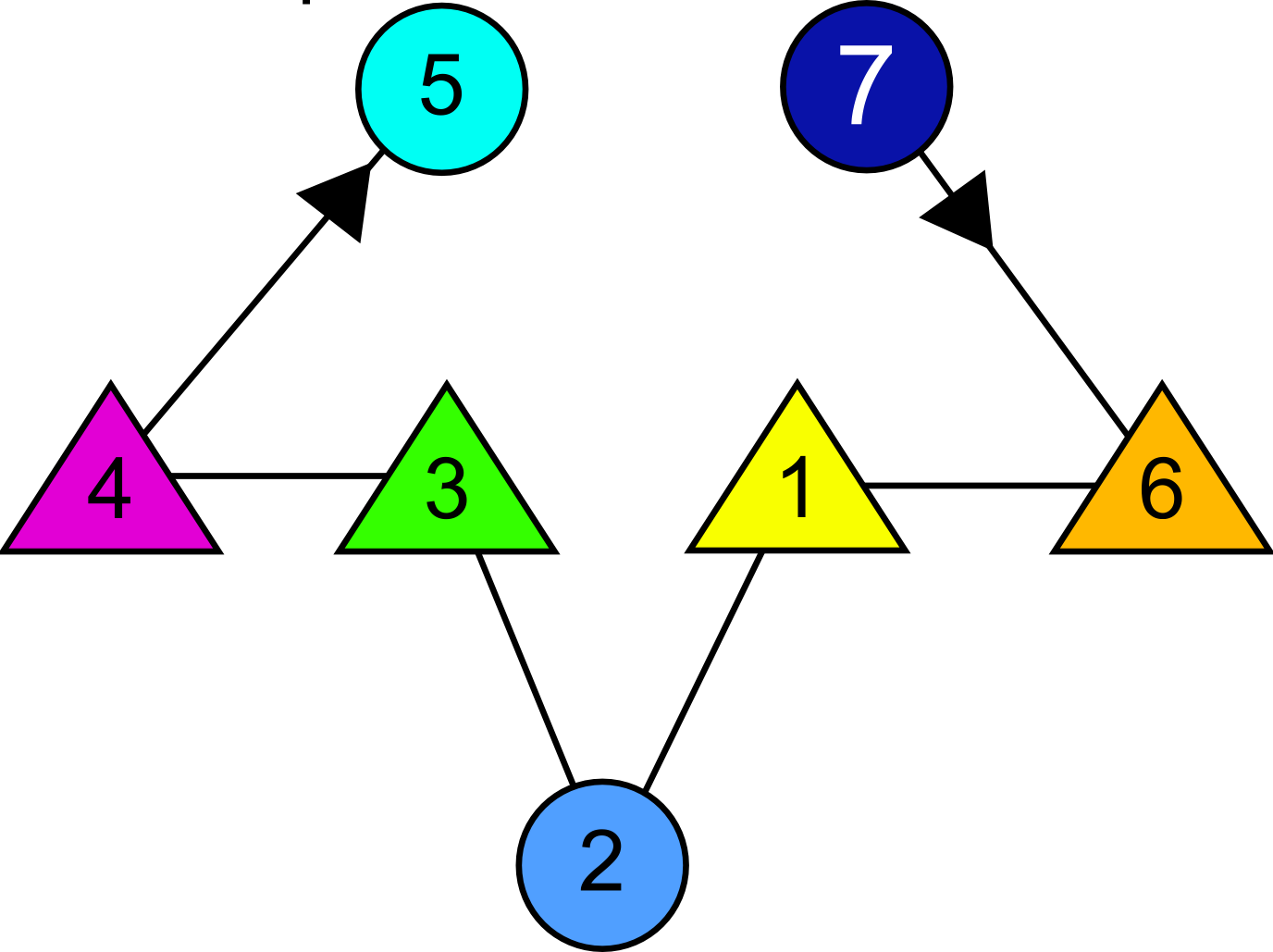 | 2 structures: 1zrn#, 1a8i# |
| 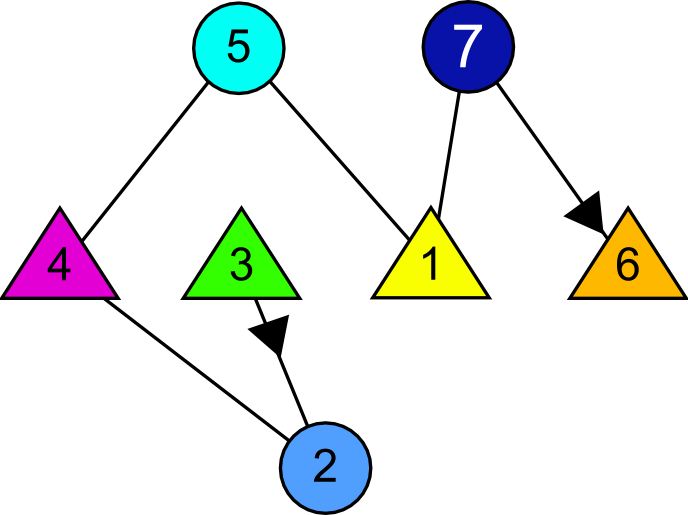 | 2 structures: 1gpjA2*, 1n2zA# |
| 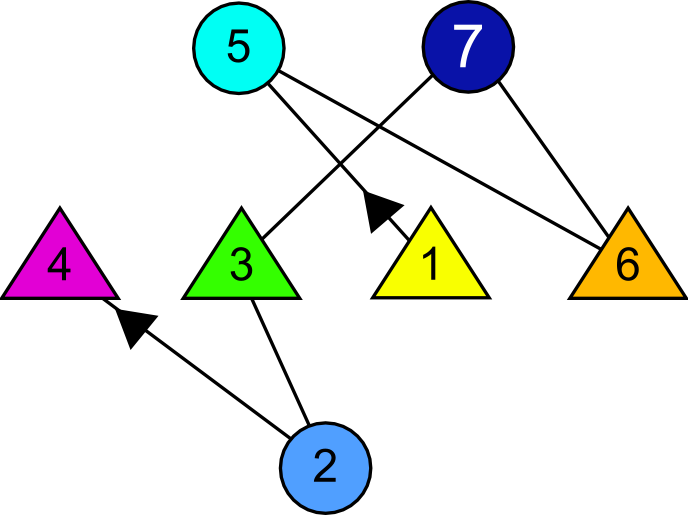 | 2 structures: 1j8fA*,1o6zA1* |
| 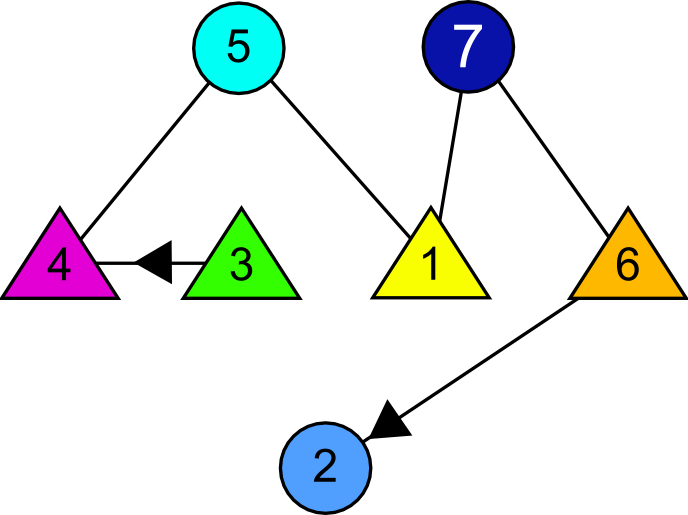 | 2 structures: 1hqsA, 1qkiA1* |
| 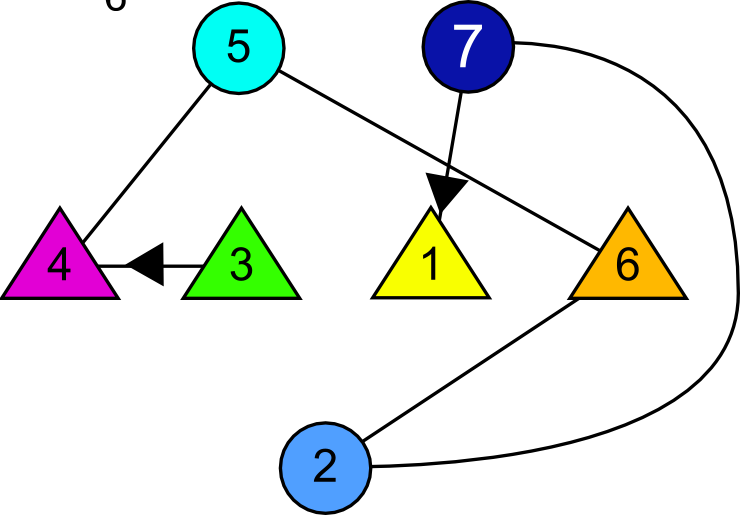 | 1 structure: 1vhyA2 (unknown function in CATH) |
| 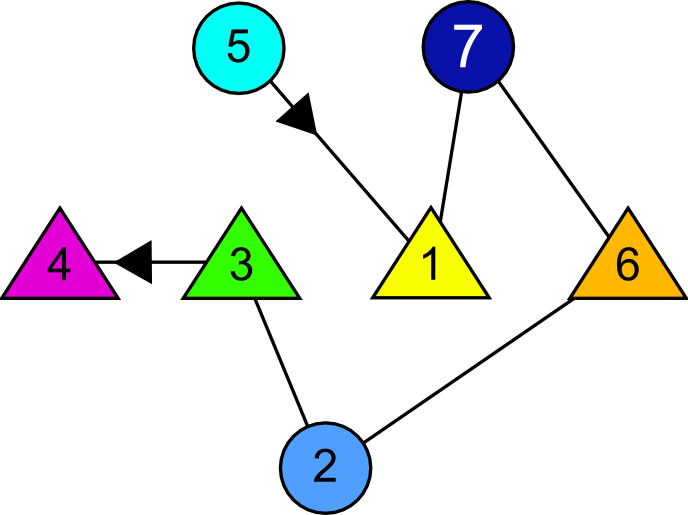 | 1 structure: 1n0wA* |
| 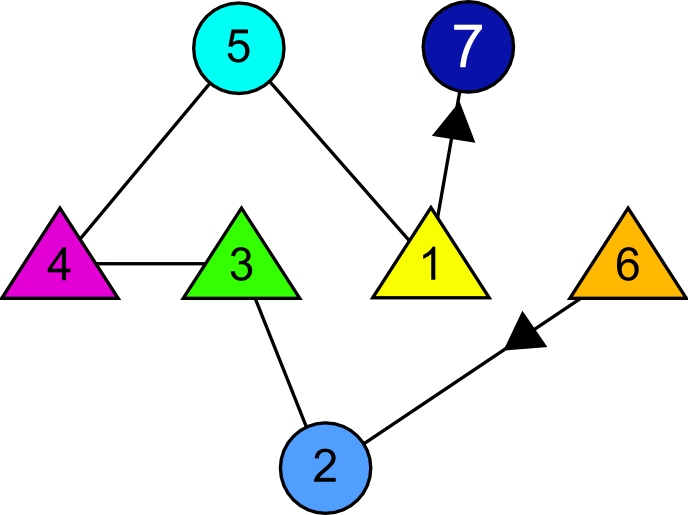 | 1 structure: 1vhvA |
| 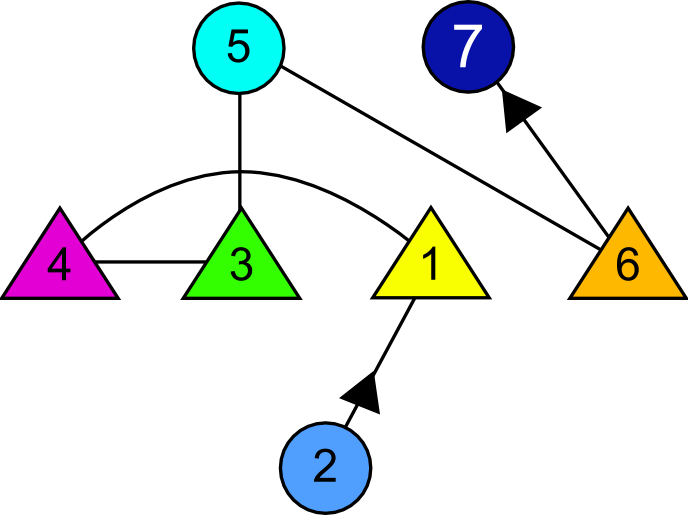 | 1 structure: 1qdeA# |
| 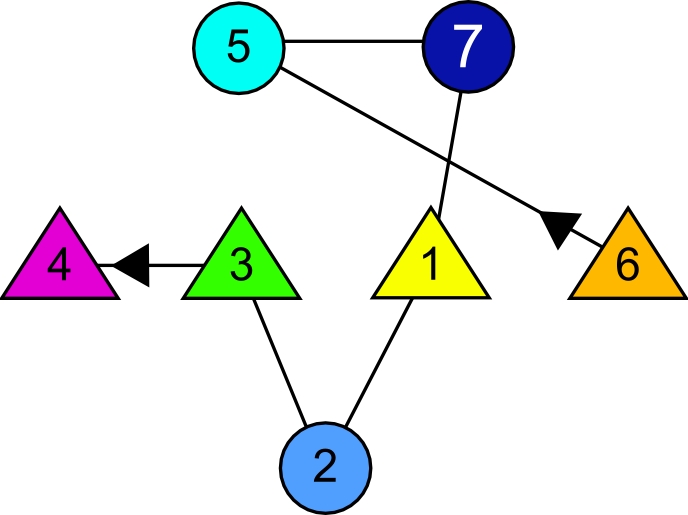 | 1 structure: 1v1aA* |
| 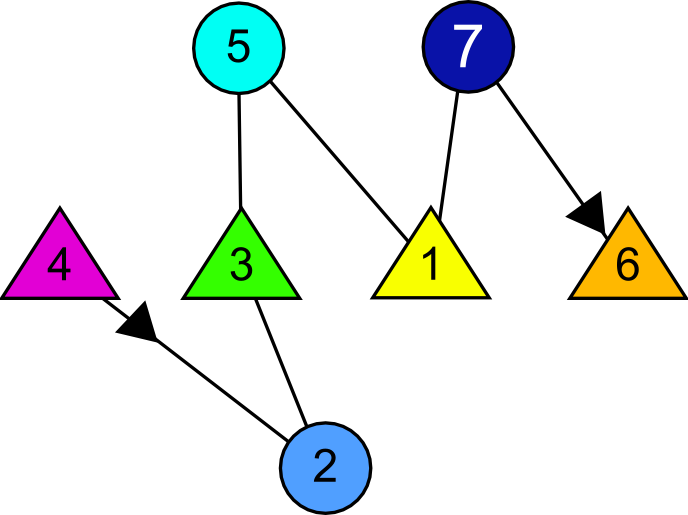 | 1 structure: 1lv7A |
| 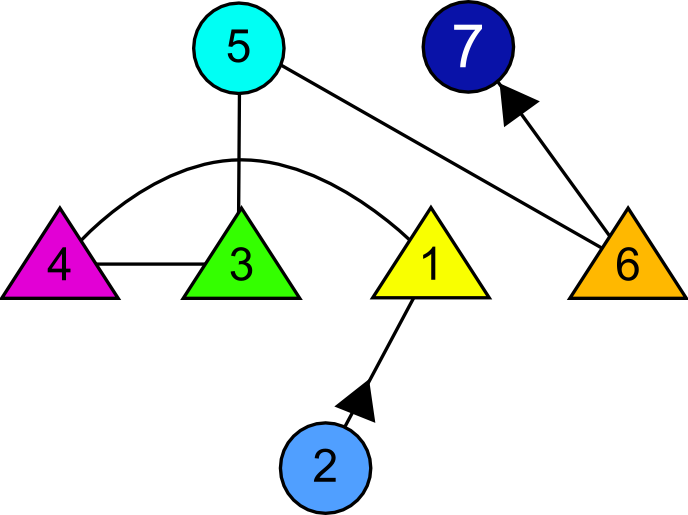 | 1 structure: 1gkuB1* |
| 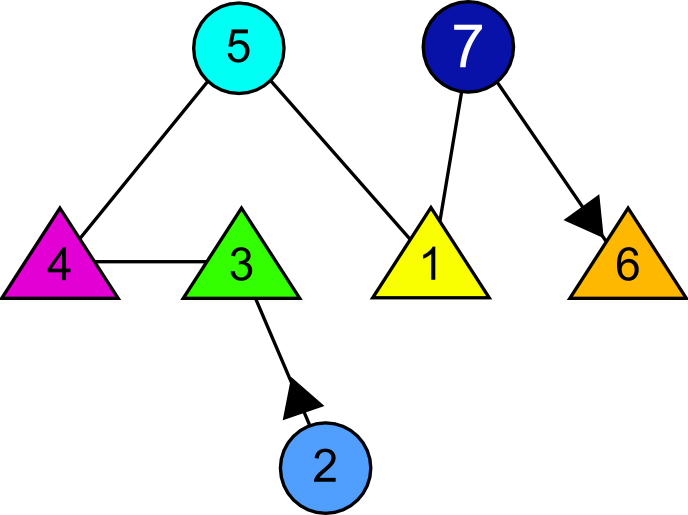 | 1 structure: 1ni4B1# |
| 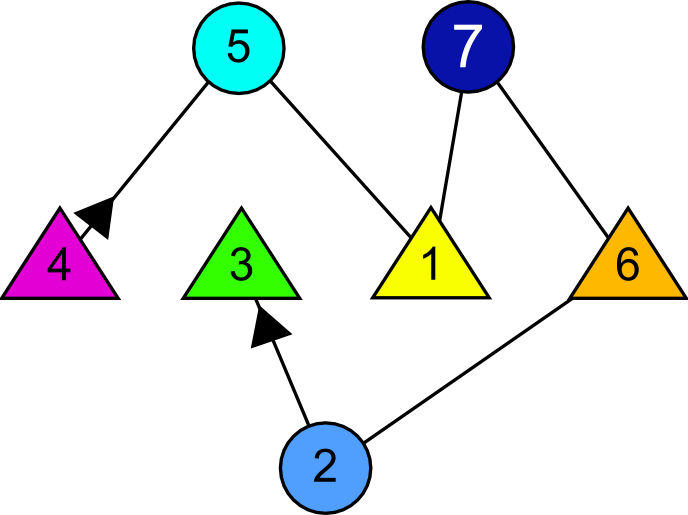 | 1 structure: 1fpzA |
| 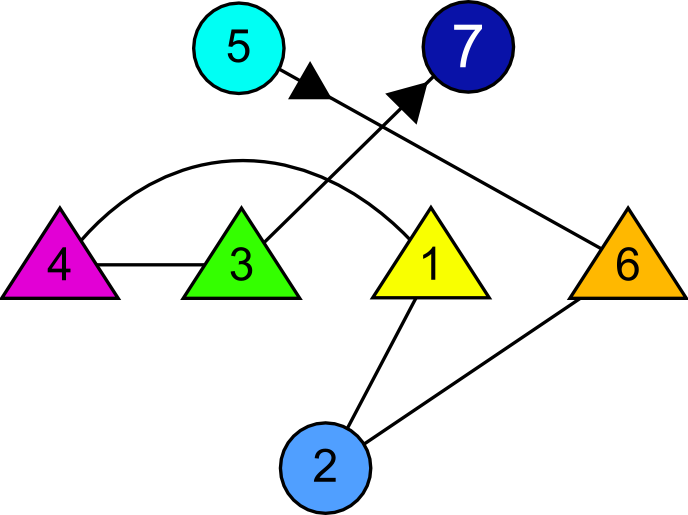 | 1 structure: 1e8cA1 |
| 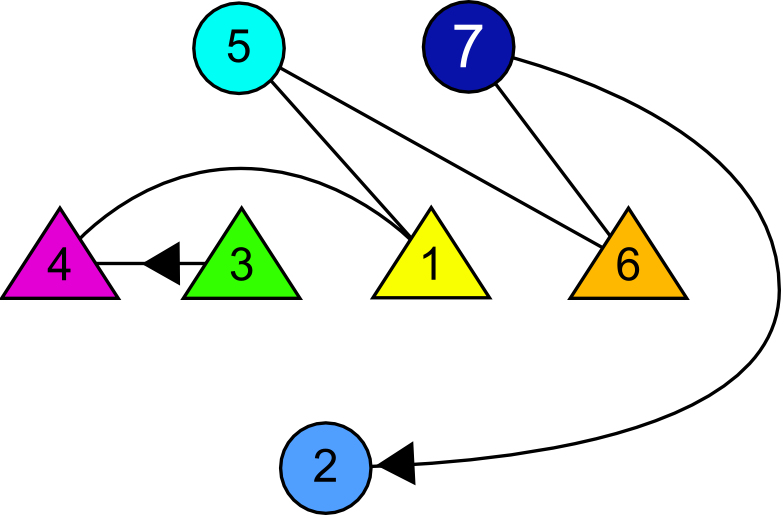 | 1 structure: 1tltA1* |
| 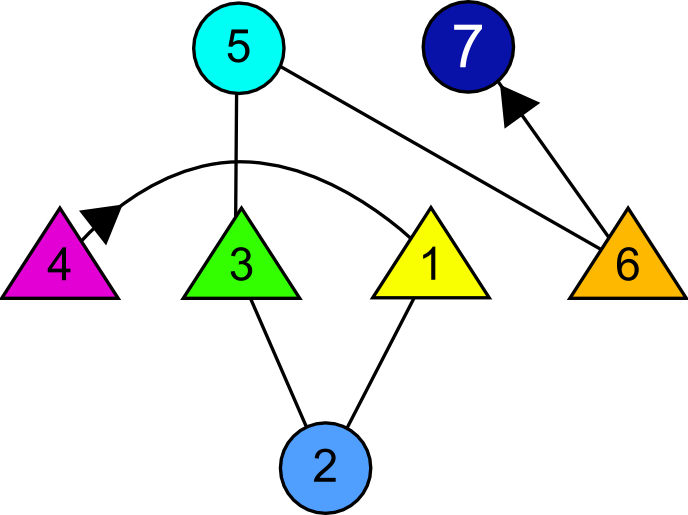 | 1 structure: 1oi7A2# |
| 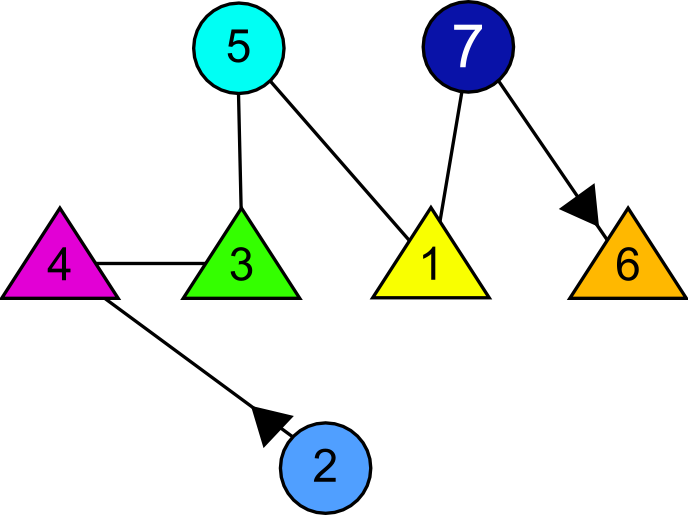 | 1 structure: 1qp8A1* |
| 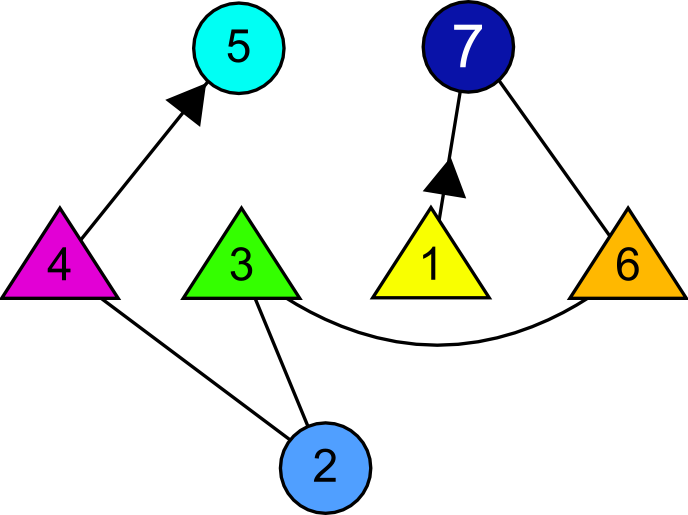 | 1 structure: 1c7qA# |
| 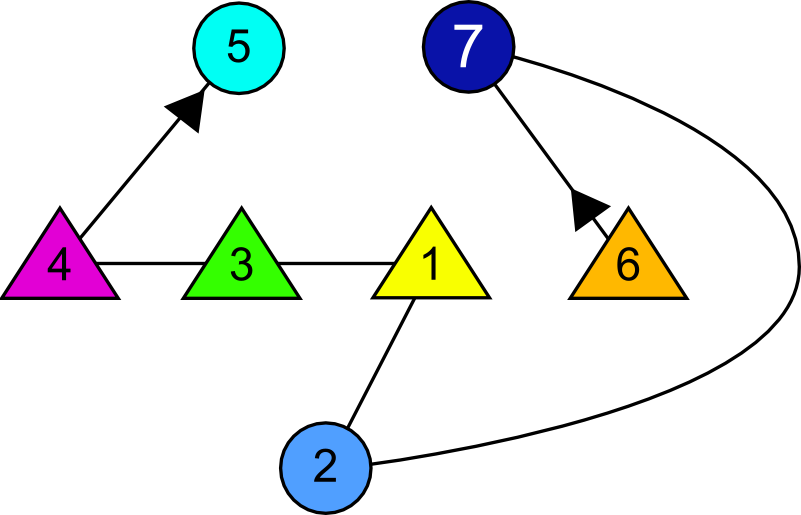 | 1 structure: 1n57A# |
| 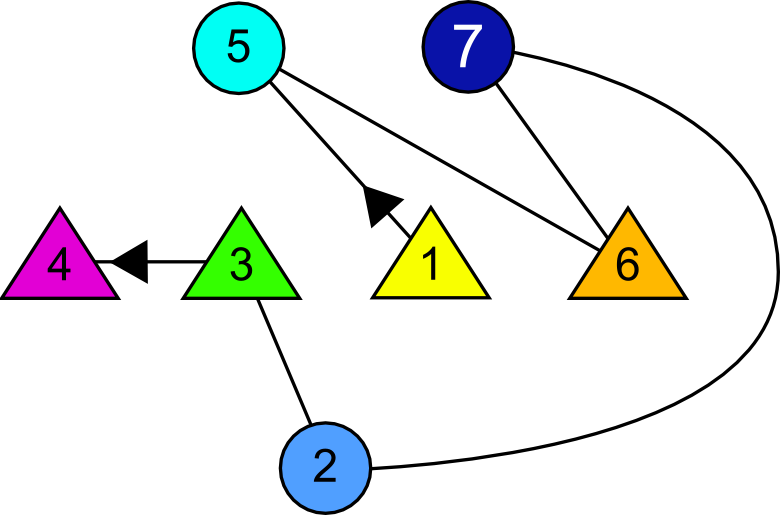 | 1 structure: 1l7dA1* |
| 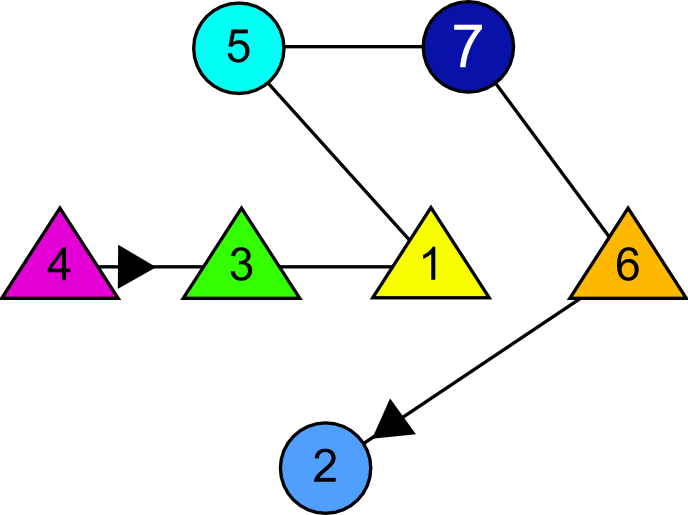 | 1 structure: 1ne7A# |
| 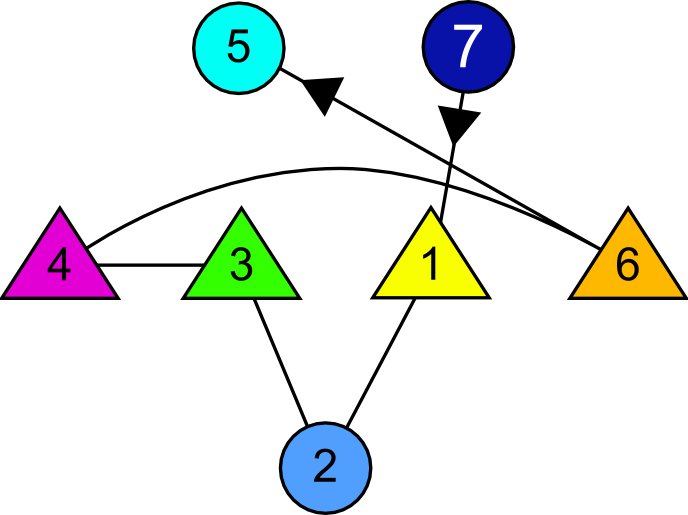 | 1 structure: 1u0tA |
| 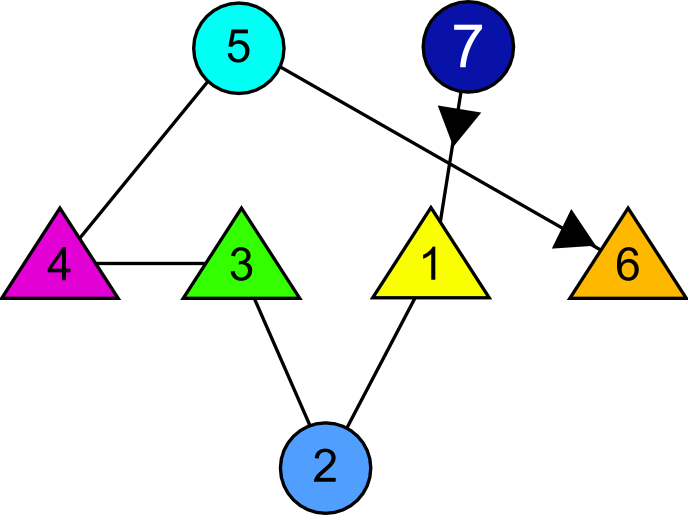 | 1 structure: 1ig0A2 |
| 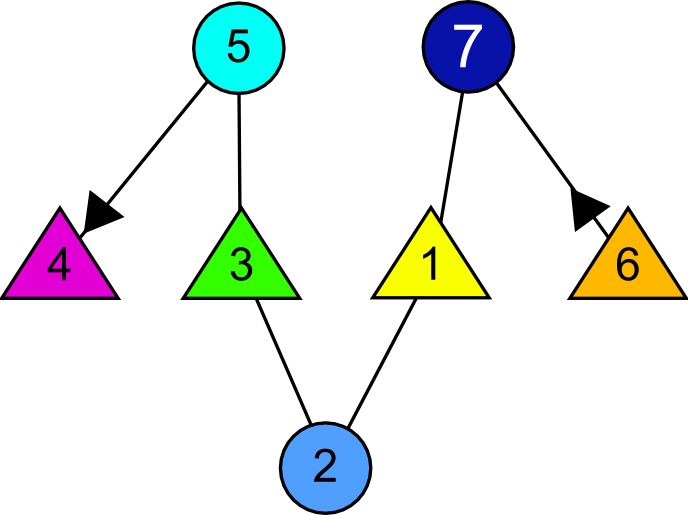 | 1 structure: 1v4vA* |
| 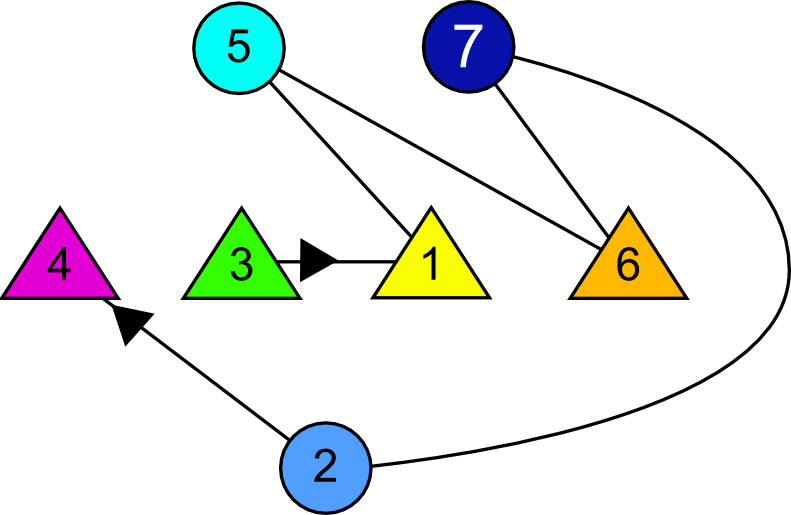 | 1 structure: 1vknA1* |
| 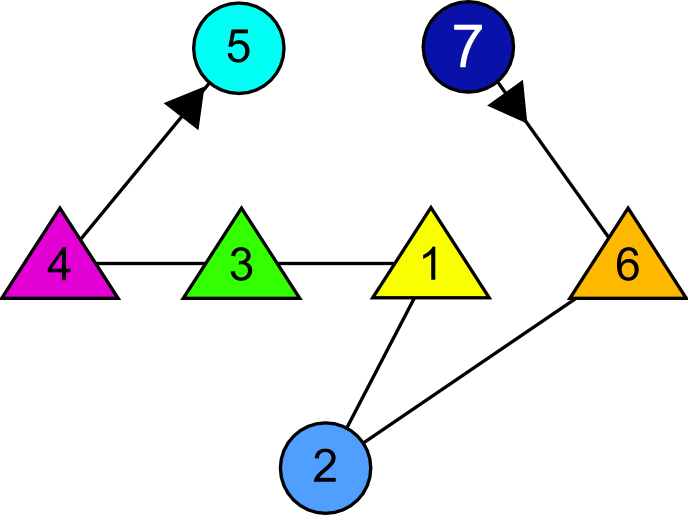 | 1 structure: 1u0jA |
| 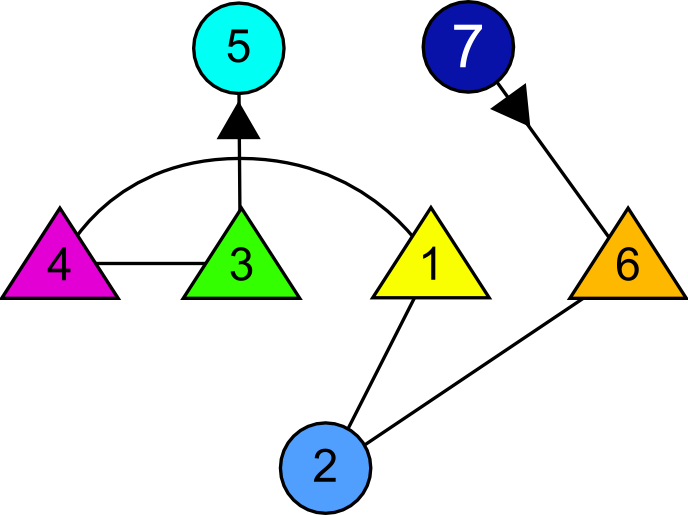 | 1 structure: 1ekwA# |
| 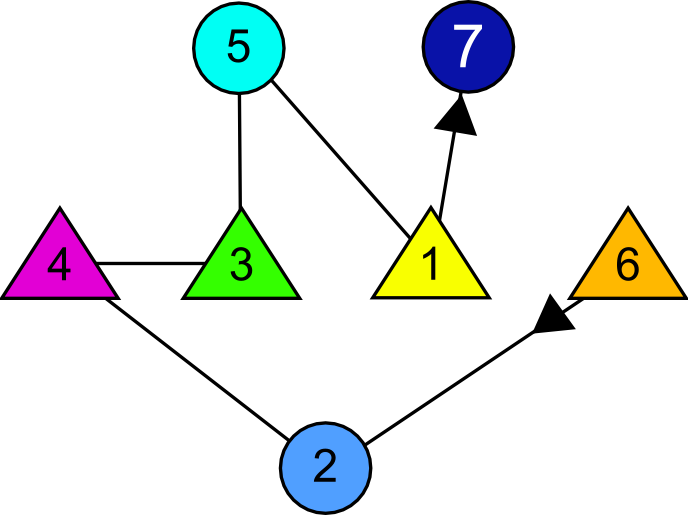 | 1 structure: 1cbf |
| 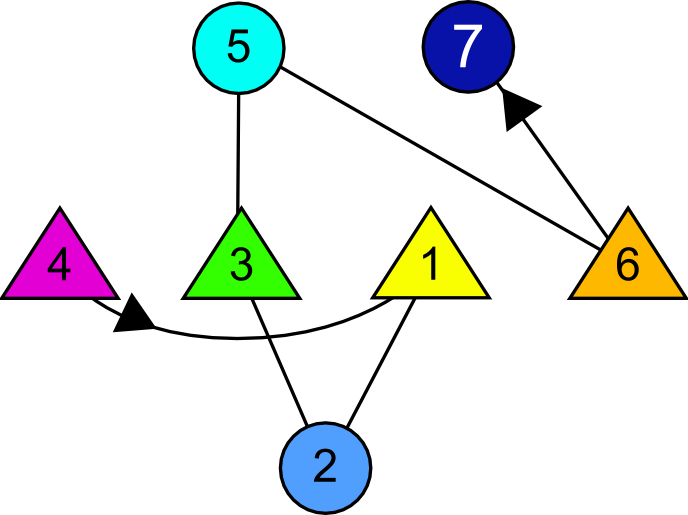 | 1 structure: 1m2zA |
| 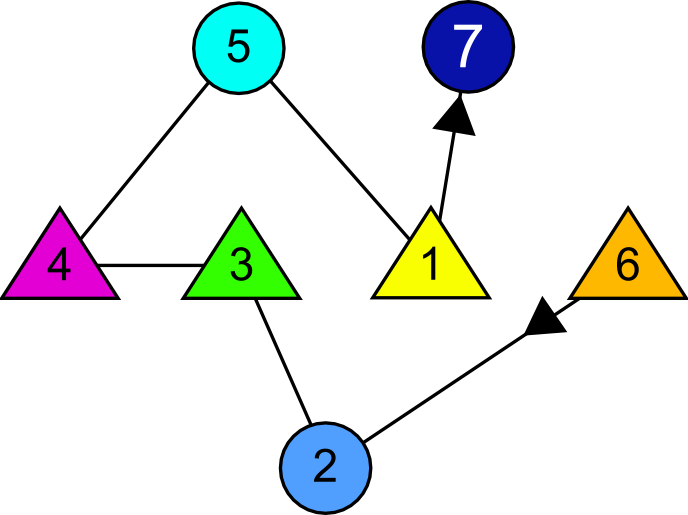 | 1 structure: 1t64A |
| 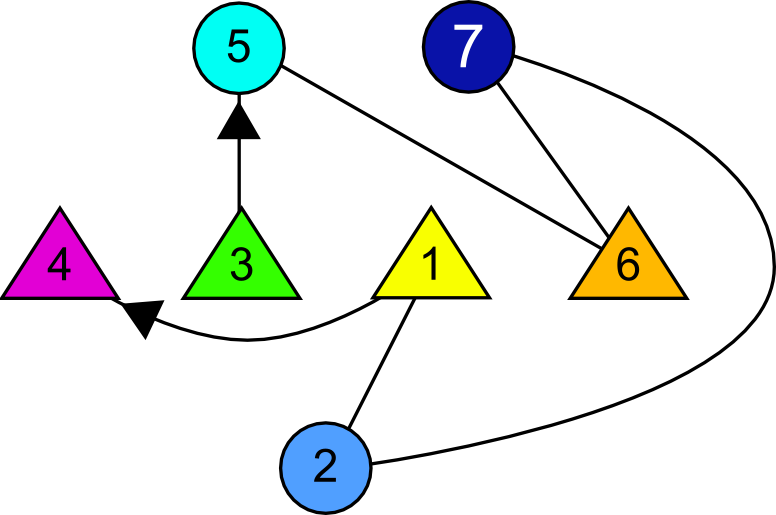 | 1 structure: 1ufoA |
| 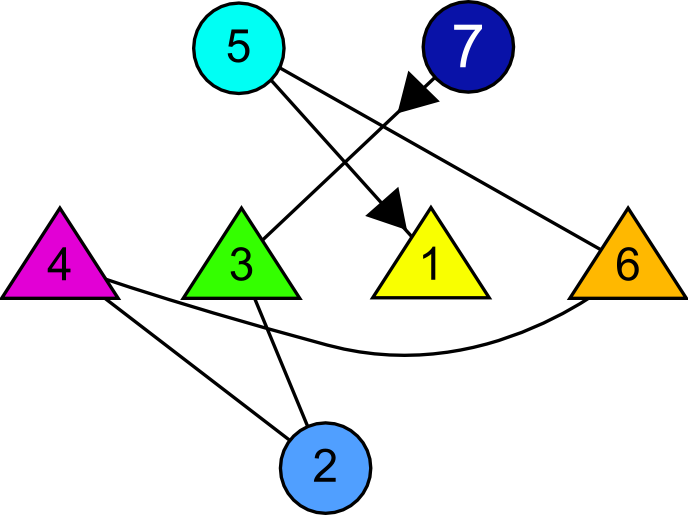 | 1 structure: 1q52A |
| 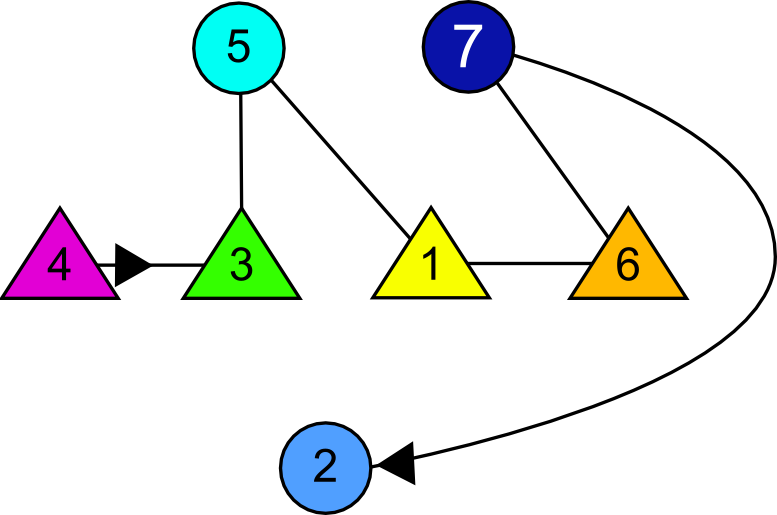 | 1 structure: 1eluA |
| 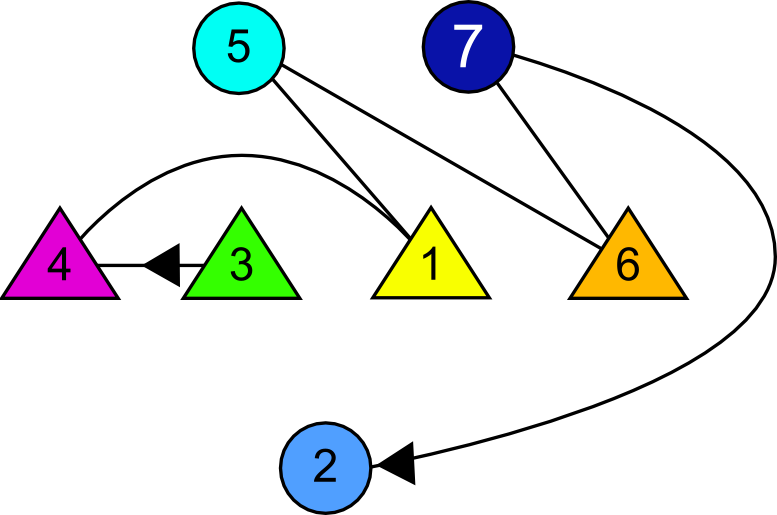 | 1 structure: 1su4A2 |
| 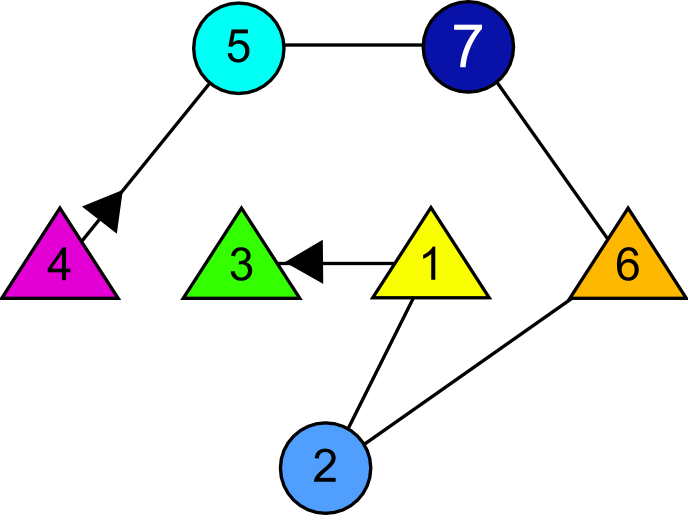 | 1 structure: 1cs1A |

**REFERENCES**

1. Chandonia JM, Walker NS, Lo Conte L, Koehl P, Levitt M, Brenner SE: **ASTRAL compendium enhancements**. *Nucleic Acid Research* 2002, **30**:264-267.

2. Murzin AG, Brenner SE, Hubbard T, Chothia C: **SCOP: a structural classification of proteins database for the investigation of sequences and structures**. *J Mol Biol* 1995, **247**:536-540.

3. Kabsch W: **A solution for the best rotation to relate two sets of vectors**. *Acta Cryst* 1978, **A32**:922-923.

4. Frishman D, Argos P: **Knowledge-based secondary structure assignment**. *Proteins* 1995, **23**:566-579.

5. Berman HM, Westbrook J, Feng Z, Gilliland G, Bhat TN, Weissig H, Shyndyalov IN, Bourne PE: **The Protein Data Bank**. *Nucleic Acid Research* 2000, **28**:235-242.

6. Novotny M, Madsen D, Kleywegt GJ: **Evaluation of protein fold comparision servers**. *Proteins* 2004, **54**:260-270.

7. Orengo CA, Michie AD, Jones S, Jones DT, Swindells MB, Thornton JM: **CATH - A Hierachic Classification of Protein Domain Structures**. *Structure* 1997, **28**(1):1093-1108.

8. Kabsch W, Sander C: **Dictionary of protein secondary structure: Pattern recognition of hydrogen-bonded and geometrical features**. *Biopolymers* 1983, **22**:2577-2637.

9. Dror O, Benyamini H, Nussinov R, Wolfson HJ: **Multiple structural alignment by secondary structures: algorithm and applications**. *Protein Science* 2003, **12**(11):2492-2507.

10. Vesterstrøm J, Taylor WR: **Flexible secondary structure based protein structure comparison applied to the detection of circular permutation.** *Journal of Computational Biology* 2006(13):43-62.

11. Rossmann MG, Moras D, Olsen KW: **Chemical and biological evolution of a nucleotide-binding protein**. *Nature* 1974, **250**:194-199.

Vesterstrøm, J. and Taylor, W.R. (2006) Flexible secondary structure based protein structure comparison applied to the detection of circular permutation., *Journal of Computational Biology*, 43-62.
